# The Potential Application of Prebiotic *Stevia rebaudiana* in Aquaculture and Animal Feed

**DOI:** 10.1155/anu/3858421

**Published:** 2025-08-28

**Authors:** Akram Ismael Shehata, Shengkang Li, Mayada Alhoshy, Hafez A. H. Mabrouk, Ghada R. Sallam, Mohamed A. Al-Absawey, Islam I. Teiba, Kumbukani Mzengereza, Walied M. Fayed, Yusuf Jibril Habib, Mohammed F. El Basuini

**Affiliations:** ^1^Guangdong Provincial Key Laboratory of Marine Biology, Shantou University, Shantou, Guangdong 515063, China; ^2^Institute of Marine Sciences, Shantou University, Shantou, Guangdong 515063, China; ^3^Department of Animal and Fish Production, Faculty of Agriculture (Saba Basha), Alexandria University, Alexandria 21531, Egypt; ^4^Independent Researcher, Alexandria, Egypt; ^5^National Institute of Oceanography and Fisheries (NIOF), Cairo, Egypt; ^6^Faculty of Agriculture, Tanta University, Tanta 31527, Egypt; ^7^Department of Fisheries and Aquatic Science, Private Bag 201, Mzuzu University, Mzuzu, Malawi; ^8^Department of Medical Analysis, Faculty of Applied Science, Tishk International University, Erbil, Iraq; ^9^Faculty of Desert Agriculture, King Salman International University, South Sinai, Sinai, Egypt

**Keywords:** aquaculture, functional ingredients, immunoantioxidant, natural feed additives, phytogenic compounds, *Stevia rebaudiana*, sustainable animal production

## Abstract

Aquaculture and animal producers are increasingly exploring natural additives such as *Stevia rebaudiana* for their health-promoting and sustainability-enhancing roles. Known primarily as a sweetener, *S. rebaudiana* also contains bioactive compounds, such as stevioside and rebaudioside A (Reb A), which exhibit antibacterial, antioxidant, immunomodulatory, and metabolic benefits. Recent studies suggest that these compounds may also exert prebiotic-like activities by modulating the gut microbiota, promoting the growth of beneficial bacterial populations (e.g., *Lactobacillus*, *Bifidobacterium* spp.), and improving intestinal integrity, thereby contributing to enhanced nutrient absorption and immune competence. This review highlights the potential of *S. rebaudiana* in animal feed and aquaculture, demonstrating its ability to enhance growth performance, strengthen immune systems, regulate fat metabolism, and improve overall animal health. Its role as a functional feed ingredient is further emphasized by its capacity to reduce oxidative stress and enhance product quality in both aquatic and terrestrial species (e.g., carp, mullet, tilapia, shrimp, laying hens, broiler, and goats), all of which align with established markers of prebiotic efficacy. By incorporating *S. rebaudiana* into animal and aquaculture diets, producers may not only improve animal productivity and welfare but also address sustainability challenges by reducing reliance on synthetic additives and antibiotics. Thus, *S. rebaudiana* represents a promising, multifunctional ingredient with potential prebiotic activity, supporting both animal health and environmentally responsible agricultural practices.

## 1. Introduction

Animal nutritionists are increasingly interested in high-performance alternative feeds, notably plant additions rich in phytochemicals. These plant-derived bioactive compounds exhibit antiviral, antioxidant, anti-inflammatory, and immune-boosting properties [1–3]. The application of these compounds aims to improve feed digestion, enhance immunity, and increase resistance to oxidative stress and inflammation, thereby improving feed efficiency and growth in fish and other farm animals [4–7]. With increased concern over antibiotic resistance and chemical residues in aquaculture, natural feed additives are gaining momentum [8, 9]. Among these, *Stevia rebaudiana*, known for its intense sweetness and functional compounds, emerges as a unique candidate with dual roles in palatability and health modulation [10].

Integrating by-products into animals' diets is a key strategy for increasing profitability and supporting environmental sustainability. Studies have demonstrated that incorporating *Stevia* leaves as an immunostimulant can enhance the immune response in fish [11, 12], positively influence intestinal microorganisms, and enhance the digestive enzyme activity [13, 14]. Additionally, *Stevia* enhances nutrient absorption and assimilation [15] and improves diet utilization by increasing feed attractiveness for aquatic animals, ultimately leading to better growth performance [16].

Originally known as *Eupatorium rebaudianum*, the sweet herb of Paraguay was identified by Bertoni, who renamed it *S. rebaudiana* Bertoni [17]. Currently, approximately 90 varieties of *S. rebaudiana* are cultivated worldwide [18]. The bulk density of *Stevia* is reported to be 0.443 g/mL, with water absorption capacity, fat absorption capacity, and emulsification value measured at 4.7, 4.5, and 5 mL/g, respectively. Its swelling ability and solubility are 5.012 and 0.365 g/g, respectively, with a pH of 5.95 [19]. *Stevia* leaves are rich in bioactive compounds, including steviosides, flavonoids, and polyphenols (PPs), which possess strong antioxidant properties. The leaves contain at least 100 phytonutrients [10]. Studies, such as those by Jawad et al. [20], highlight the potential of these compounds to reduce oxidative stress and support health in aquaculture species. In omnivorous fish, such as mullet, tilapia, and carp, the digestive tract is well-suited to plant-based supplements rich in glycosides and PPs [16, 21, 22].

*Stevia* serves both as a functional feed additive and a natural sweetener, enhancing feed palatability and health outcomes. *S. rebaudiana* is the most well-documented species in the genus *Stevia*, belonging to the Asteraceae family [23, 24]. The *Stevia* genus includes around 150 species, including *Stevia dianathoidea*, *Stevia phlebophylla*, *Stevia anisostemma*, *Stevia bertholdii*, *Stevia crenata*, *Stevia enigmatica*, *Stevia eupatoria*, *Stevia lemmonii*, *Stevia micranatha*, *Stevia ovata*, *Stevia plummerae*, *Stevia rebaudiana*, *Stevia salicifolia*, *Stevia serrata*, and *Stevia viscida*, with *S. rebaudiana* being the sweetest [25]. *Stevia* leaves, particularly those of *S. rebaudiana*, contain multiple diterpene glycosides, which are extracted for use as natural, non-nutritive, and noncaloric sweeteners [24, 26, 27]. In addition to its sweetening properties, *Stevia* leaves contain proteins, fiber, essential minerals (Fe, P, Ca, K, Na, Mg, Zn), rutin, antioxidants, phenols, β-carotene, vitamin C, and oils, along with 53 other constituents [28].

*Stevia* leaf meal may offer nutritional benefits for fish due to its bioactive compounds and antioxidants. These leaves contain phenolic compounds, such as chlorogenic acids and flavonoids, which exhibit strong antioxidant properties, supporting cellular health. The glycosides in *Stevia* leaves, while non-nutritive, may help balance glucose levels and promote metabolic health in fish, potentially enhancing feed efficiency and immunity [29]. By-products of *Stevia*, including stems, seeds, flowers, and leaves not selected for industrialization, are processed into animal feed or fertilizers [30]. Studies have shown that adding natural or artificial sweeteners to ruminants' diets can increase feed intake and average daily gain [31, 32]. For decades, many countries, particularly in Latin America and East Asia, have used dried *Stevia* leaves and aqueous extracts as sweeteners [33]. These components illustrate the potential of *S. rebaudiana* as a functional ingredient in fish diets, both for its nutritional contributions and bioactive properties. Further research is needed to explore the efficacy of *Stevia* in enhancing fish growth and health outcomes, as current studies on its use in aquaculture are still limited [21, 22, 34].

*Stevia* leaves contain eight glycosides derived from the tetracyclic diterpene steviol. Stevioside, the most prevalent sweetening component, accounts for 5%–10% of the dry leaf weight and is 300 times sweeter than sucrose [35]. Other glycosides include rebaudioside A (Reb A) (2%–4% of dry weight), rebaudioside B and C (up to 2%), rebaudioside D and E (0.4%–0.7%), as well as dulcoside A and steviolbioside, which have sweetness levels ranging from 50 to 450 times that of sucrose [35, 36]. Takahashi et al. [37] found that *Stevia* stem extract contains active compounds that reduce histamine activity, with the antioxidant power of *Stevia* stem being higher than that of its leaves and even green tea extract. Seidavi et al. [38] included *Stevia* in a list of valuable plants containing bioactive compounds for use as phytogenic feed additives. While *Stevia* stems have high crude fiber and low metabolizable energy content, limiting their nutritional value for monogastric animals, they may serve as a supplemental energy source or sweet energy diluent in diets [39].

Therefore, the purpose of this review is to present a comprehensive and up-to-date synthesis of the potential application of *S. rebaudiana* as a functional feed additive in aquaculture and animal nutrition. This review highlights the phytochemical composition and health-promoting properties of *S. rebaudiana*, with particular emphasis on its antioxidant, antimicrobial, immunomodulatory, and metabolic effects. Special attention is given to the emerging prebiotic potential of *S. rebaudiana*, including its ability to modulate gut microbiota, enhance nutrient absorption, and support intestinal and immune health in aquatic and terrestrial animals. Additionally, this review explores the role of *S. rebaudiana* in enhancing feed palatability, growth performance, and sustainability, while also discussing its safety profile and limitations. By integrating recent findings, this work aims to establish a scientific basis for the use of *S. rebaudiana* in promoting animal health and improving the sustainability of animal and aquaculture production systems.

## 2. Nutritional Composition of *Stevia* Leaves

### 2.1. Macronutrients

Macronutrients are essential nutrients required in large amounts for energy, growth, metabolic efficiency, and cellular functions. They include proteins, which provide amino acids for tissue repair and metabolism; carbohydrates, the primary energy source, encompassing both digestible sugars and dietary fibers; and lipids, which supply energy and fatty acids that support cellular structure (Table 1).

#### 2.1.1. Proteins and Amino Acids

*Stevia* leaves contain approximately 16% crude protein, while the stems hold about 6.7% [39]. The average energy content of dried *Stevia* plants is 2.7 kcal/g, with protein content (6.2%–20.4% DW) varying with cultivar and processing. At the upper end, it is comparable to alfalfa or duckweed, supporting its use as a protein extender in omnivorous fish diets [19, 41, 44, 47, 48]. Although *Stevia* is not a primary source of protein, its amino acid profile, based on plant composition analyses, can potentially synergize with protein-rich ingredients in aquatic feeds to support metabolism, tissue repair, and growth [49]. Its protein content is comparable to other conventional protein sources used in aquafeeds, making it a valuable supplemental ingredient for meeting fish nutritional needs [50]. Research analyzing *Stevia* plant material has identified several essential amino acids, including lysine and leucine, as well as a range of nonessential amino acids, reinforcing *Stevia*'s potential suitability as a dietary supplement [51]. Rafiq et al. [52] identified eight essential amino acids, glutamic acid, aspartic acid, lysine, serine, isoleucine, alanine, proline, and tyrosine, while Abou-Arab et al. [41] expanded this list to include 17 amino acids, comprising nine essential and eight nonessential amino acids, with tryptophan being the only amino acid absent. These findings underscore *Stevia*'s compositional potential as a complementary ingredient to enhance the amino acid balance in fish diets. However, in vivo studies are needed to determine its bioavailability, species-specific effects, and optimal inclusion levels in aquafeed formulations.

#### 2.1.2. Lipids Fatty Acids

Dried *Stevia* leaves contain lipids ranging from 1.9 to 5.6 g/100 g [43]. Manish et al. [44] found that *Stevia* leaves are a good source of protein, carbohydrates, and inorganic minerals, but their low-fat content indicates that they are not a significant source of oil. They identified six fatty acids (g/100 g): palmitic acid (27.51), palmitoleic acid (1.27), stearic acid (1.18), oleic acid (4.36), linoleic acid (12.4), and linolenic acid (21.59). The study revealed a high content of palmitic acid in *Stevia* leaves, while stearic acid had the lowest content among the fatty acids. Atteh et al. [39] demonstrated that *Stevia* leaves contain the following fatty acids (g/100 g): palmitic acid (29.5), linolenic acid (32.6), linoleic acid (16.8), oleopalmitic acid (3.0), stearic acid (4.0), and oleic acid (9.9). Environmental and processing factors may explain variations in fatty acid composition. Despite its low-fat content, *Stevia* serves as a source of unsaturated fatty acids beneficial in aquafeeds. In particular, linoleic and linolenic acids, classified as essential fatty acids for many aquatic species, play crucial roles in maintaining membrane integrity, regulating inflammatory responses, and enhancing immune and antioxidant functions [53]. Although *Stevia*'s lipid content may not fully satisfy the essential fatty acid requirements of fish, its composition can still contribute to the overall fatty acid profile of aquafeeds and support basic physiological processes. These data on the chemical composition of Stevia leaves encourage their use as an economical nutritional alternative to fish feed ingredients.

#### 2.1.3. Carbohydrates

*Stevia* leaves contain carbohydrates predominantly in the form of dietary fibers, including cellulose, hemicellulose, and lignin. These indigestible fibers account for 35.2%–72.6% of the plant's dry weight [48]. Digestible carbohydrates, such as soluble sugars and starches, are relatively low, typically less than 5% of the total carbohydrate content. The high fiber content of *Stevia* limits its role as a primary energy source, particularly for monogastric animals like fish, but it provides significant prebiotic benefits by supporting gut health and promoting the growth of beneficial microflora [54, 55].

*Stevia*'s carbohydrate profile includes both soluble and insoluble fibers, which are essential for digestive health. Insoluble fibers, such as cellulose and lignin, enhance gut motility and promote chyme movement, while soluble fibers, including hemicellulose, are fermented in the gut to support microbial proliferation [56]. Studies have shown that dietary fiber acts as a prebiotic, fostering the growth of beneficial bacteria such as *Bifidobacteria* and *Lactobacillus* [57]. High-fiber diets also encourage the development of fiber-degrading bacterial populations, further improving gut efficiency [58].

The average crude fiber content in dried *Stevia* leaves ranges from 13.56% to 18.5% [41, 48]. On a dry, fat-free basis, crude fiber content is reported as 6.8% in leaves and 45.4% in stems [39]. Sánchez-Muniz [59] further identified that *Stevia* leaves contain 28.61–29.12/100 g of total dietary fiber, with 70.02%–87.79% consisting of insoluble fiber, such as acid detergent lignin (2.28%–8.98%), neutral detergent fiber (18.11%–19.29%), and acid detergent fiber (14.16%–17.77%). Hemicellulose content ranges from 1.51% to 3.96%, and cellulose accounts for 8.79%–11.78%. These fibers contribute to plant cell structure and provide functional benefits, such as promoting bowel movements and improving digestive health in both humans and animals. Energy values for *Stevia* vary depending on the processing method, ranging from 265.9 to 384.2 kcal/100 g [19, 60]. Digestible carbohydrates, primarily in the form of starch, constitute a minor fraction, with reported levels of 69.9% in certain plant parts. Additionally, *Stevia* leaves contain small amounts of antinutritional factors, such as oxalic acid (0.02 g), which should be considered when formulating diets [61].

*Stevia* also contains fructooligosaccharides (FOSs), which are present at levels of 4.6% in roots and 0.46% in leaves. These compounds have been shown to regulate lipid metabolism [62] and help control blood sugar levels [63]. Furthermore, the high crude fiber content of *Stevia* stems, combined with their low metabolizable energy value, suggests limited use as a primary feed ingredient for monogastric animals but highlights their role as a potential functional component in aquafeeds to promote gut health and stability [39]. In aquaculture, *Stevia*'s dietary fibers can improve pellet stability, reducing nutrient loss in aquatic environments. The insoluble fiber fraction of *S. rebaudiana* primarily enhances intestinal peristalsis by adding bulk to the digesta and stimulating mechanoreceptors in the intestinal wall, thereby promoting gastrointestinal motility and reducing transit time [64]. In contrast, the soluble fiber fraction exerts prebiotic effects by being selectively fermented by beneficial gut microbiota, such as Lactobacillus and Bifidobacterium species, resulting in the production of short-chain fatty acids (SCFAs), including acetate, propionate, and butyrate [65]. These SCFAs play crucial roles in maintaining gut barrier integrity, modulating immune responses, and suppressing the growth of pathogenic bacteria [66]. Through these complementary mechanisms, dietary fiber from *Stevia* contributes to intestinal health and microbial balance, supporting its potential as a functional ingredient in aquafeed formulations aimed at improving digestive efficiency and sustainability.

### 2.2. Micronutrients

*Stevia* contains a variety of micronutrients that enhance its nutritional profile. Stevia is rich in vitamins, including vitamins A, C, and B-complex vitamins like thiamine and riboflavin [67]. A previous study showed that *Stevia* contains water-soluble vitamins such as vitamin C and vitamin B complex, with folic acid being the major compound in leaves, followed by vitamin C [68]. *Stevia* is also a source of essential minerals, including potassium, phosphorus, magnesium, calcium, copper, sodium, iron, silicon, chromium, cobalt, manganese, zinc, and selenium [69]. These minerals are beneficial for bone formation, the immune system, and enzymatic processes, supporting the excretory system, oxygen utilization, and energy metabolism [70].

The dried leaves powder had average amounts of 470.33 mg/100 g of calcium, 7.64 mg/100 g of iron, and 27.00 mg/100 g of phosphorus, while the fresh juice contained 432.00 mg/100 g of calcium, 0.98 mg/100 g of iron, and 26.16 mg/100 g of phosphorus, as shown in Table 2. According to Abou-Arab et al. [41], *Stevia* plants are a good source of carbohydrates (61.93% DW), protein (11.41% DW), crude fiber (15.52% DW), and minerals (K = 21.15; Ca = 17.7; Na = 14.93; M = 3.26 mg/100 g DW; Cu = 0.73; M = 2.89; Fe = 5.89; and Z = 1.26 mg/100 g DW). *Stevia* leaves also contain minerals such as K, Mg, S, Na, P, Cu, Co, Fe, Mn, Zn, Se, and Mo [73].

#### 2.2.1. Vitamin C, B-Complex Vitamins

Vitamins play a multifaceted role in physiological functions, particularly in combating various husbandry and physical stressors in aquaculture [74, 75]. *S. rebaudiana* leaves contain a variety of vitamins, including vitamin C and B-complex vitamins, which contribute to their nutritional qualities [67]. These vitamins complement the antioxidant, anti-inflammatory, and antimicrobial effects of other bioactive compounds in *Stevia*, enhancing its value as a functional ingredient in animal nutrition. For aquaculture, the inclusion of vitamin-rich components like *Stevia* in diets could promote stress tolerance, boost immunity, and improve growth performance in fish, making it a promising natural additive for sustainable farming practices [75].

There are 13 essential vitamins, categorized into four fat-soluble vitamins (A, D, E, and K) and nine water-soluble vitamins (eight B-complex vitamins and vitamin C). These compounds have diverse biochemical roles. Some vitamins function as hormone-like regulators of mineral metabolism (e.g., vitamin D) or as regulators of cell and tissue growth and differentiation (e.g., some forms of vitamin A). Others act as antioxidants (e.g., vitamin E and sometimes vitamins B and C). The largest group of vitamins, the B-complex vitamins, primarily function as precursors of enzyme cofactors [76].

##### 2.2.1.1. Vitamin C

Vitamins are organic micronutrient substances present in very small quantities in food but are necessary for metabolism. Gupta et al. [48] found that the juice extract of *Stevia* leaves contains a comparatively high amount of ascorbic acid (30.13 mg/100 g). Most teleost fish lack the enzyme gluconolactone oxidase, which is necessary for vitamin C biosynthesis, and thus rely on dietary supplementation [77]. *S. rebaudiana* includes vitamin C (ascorbic acid), an important antioxidant that boosts its nutritional value. Vitamin C is well-known for its ability to neutralize free radicals, which reduces oxidative stress and protects cells against reactive oxygen species (ROS) [78]. This antioxidant action not only benefits the plant by defending against environmental stressors but also contributes to the health benefits of *Stevia* in animal diets [67].

In aquaculture, a critical role of vitamin C is the synthesis of connective tissue, particularly collagen [79]. It also supports the immune system, reducing the risk of infections [80], and enhances overall growth and reproductive performance in fish [81]. Additionally, vitamin C is involved in many other metabolic functions [82, 83]. Incorporating *Stevia*, with its vitamin C content, into fish diets could improve stress tolerance, enhance resistance to infections, and support tissue repair. The presence of vitamin C in *Stevia* highlights its potential as a natural and functional feed additive for sustainable fish farming [84].

##### 2.2.1.2. Vitamin B

*S. rebaudiana* is a source of various B vitamins, including B_1_ (thiamine), B_2_ (riboflavin), and B_6_ (pyridoxine), which are essential micronutrients that play critical roles in cellular metabolism and energy production. These vitamins contribute to the overall health-promoting properties of *Stevia*, aiding in processes such as carbohydrate metabolism, red blood cell formation, and nervous system function [67]. In fish, B vitamins are crucial for growth, immunity, and physiological functions. For instance, thiamine supports energy metabolism, riboflavin contributes to enzyme function and antioxidant defense, and pyridoxine is vital for amino acid metabolism and neurotransmitter synthesis [85]. The B vitamins, such as riboflavin (B_2_) and niacin (B_3_), support energy metabolism and play crucial roles in enzymatic functions [39]. Incorporating *Stevia*, with its vitamin B content, into aquatic feeds could enhance fish health, improve nutrient utilization, and promote sustainable aquaculture practices.

##### 2.2.1.3. β-Carotene

β-carotene is a naturally occurring pigment and a precursor to vitamin A (provitamin A), belonging to the carotenoid family. It is responsible for the orange, red, and yellow colors in many fruits and vegetables. The body converts β-carotene into vitamin A, an antioxidant that helps neutralize free radicals, supports immune function, and promotes healthy vision. *S. rebaudiana* contains small amounts of β-carotene as part of its nutritional profile, though the exact concentration varies based on cultivation conditions and processing methods. Typically, its carotenoid content, including β-carotene, is present in the leaf extracts, but it is relatively minor compared to its other bioactive compounds, such as steviol glycosides. To precisely determine the levels of β-carotene in *Stevia* leaves, detailed quantitative studies are necessary [86].

Vitamin A helps cells grow and keeps the nervous system healthy, which could make fish more resistant to stress. Feed formulas could achieve extra vitamin support by adding *Stevia*. Gupta et al. [48] found bioactive compounds in the leaves of *S. rebaudiana*, with β-carotene (344.0 µg/100 g) being the main compound in the dried powder.

##### 2.2.1.4. Vitamin B_9_ (Folate)

Folic acid (C_19_H_19_N_7_O_6_), first synthesized by Pfiffner et al. [87], was identified as a crucial dietary supplement for combating diet-induced anemia in fish. Early studies demonstrated its efficacy as an antianemic agent [88]. Building on these findings, Lall and Dumas [89] explored the incorporation of folic acid into experimental diets for trout, marking a significant advancement in aquaculture nutrition. These pioneering investigations established the essential role of folic acid in preventing anemia and promoting fish health, thereby forming the foundation for its widespread application in aquaculture [90]. Folic acid plays a critical role in the normal formation of blood cells and functions as a coenzyme in one-carbon transfer processes [91, 92]. Its deficiency in fish has been linked to a range of adverse effects, including poor growth, lethargy, fragility of the caudal fin, dark pigmentation, and macrocytic anemia [93]. *S. rebaudiana* contains trace amounts of vitamin B_9_, estimated at 52.18 mg/100 g [68]. Vitamin B_9_ or folic acid is a water-soluble B vitamin that is required for DNA synthesis, repair, and methylation. Its role in promoting cellular development, division, and the creation of red blood cells is crucial for overall health and metabolic activities [94]. Folic acid is especially important in aquaculture, as it aids in nucleotide and amino acid synthesis, supporting DNA and RNA production, which is critical for cell division and fish development. A deficiency can lead to macrocytic anemia, poor growth, and impaired health.

In aquaculture, different species and stages of life have different vitamin B_9_ requirements. Salmonids should receive 1–2 mg/kg of feed to help them grow and prevent anemia [85]. Specific requirements for other species include 0.82 mg/kg for juvenile tilapia (*Oreochromis niloticus*) [95], 0.8 mg/kg for juvenile grouper (*Epinephelus* spp.) [96], and 2.62 mg/kg for juvenile abalone (*Haliotis discus*) [97]. Rainbow trout (*Oncorhynchus mykiss*) require 0.3–1.1 mg/kg diet depending on growth and tissue saturation [98], while 0.5–1.0 mg/kg optimizes hematology and growth in Nile tilapia [99]. Asian seabass (*Lates calcarifer*) thrive on 1.5 mg/100 g of diet [100]. Supplementing freshwater prawns (*Macrobrachium rosenbergii*) with 2 mg/kg of folic acid improves their antioxidant defenses, growth, and survival rates [101]. These findings show the importance of including sufficient vitamin B_9_ in aquatic organism feed to improve fish health and aquaculture efficiency. Therefore, due to its high folic acid content, *Stevia* can serve as an effective natural source of folic acid in aquatic organism feeds for both marine and freshwater fish, thereby supporting growth, health, and productivity in aquaculture.

#### 2.2.2. Minerals

*S. rebaudiana* leaves contain essential minerals, including potassium (K), calcium (Ca), magnesium (Mg), iron (Fe), and zinc (Zn), each of which plays a distinct role in supporting fish health. Potassium and calcium are crucial for osmoregulation and skeletal development in fish, while iron is essential for hemoglobin production and oxygen transport within the body [67]. *Stevia* is a mineral-rich food source, providing essential nutrients required for optimal biological processes and overall health. The leaves of *S. rebaudiana* contain significant amounts of potassium, calcium, magnesium, and sodium, all of which are vital for maintaining physiological balance and health [102].

The high potassium content reported in various studies is particularly noteworthy. However, the potassium levels reported by Abou-Arab et al. [41] appear to be lower compared to other studies, which may be attributed to differences in growth conditions, as suggested by Ramesh et al. [35]. *Stevia* leaves also contain zinc and iron, which are essential for both plant- and animal-derived diets. Zinc acts as a nonenzymatic antioxidant, helping to prevent oxidative cell damage [103]. Iron, on the other hand, is fundamental for oxygen transport in the body, and its deficiency can lead to anemia. The high iron content in *Stevia* leaves may help maintain adequate hemoglobin levels, making it a potential dietary supplement to address iron deficiency anemia, a common nutritional issue in developing countries [41]. Therefore, incorporating *Stevia* into fish diets can serve as an additional source of essential minerals, although the overall dietary composition may require adjustments to optimize feed formulations.

### 2.3. Phytochemicals

*Stevia* contains a variety of phytochemicals that contribute to its health-promoting properties. The primary bioactive compounds are steviol glycosides, including stevioside and rebaudiosides, which provide intense sweetness with minimal caloric content. Additionally, *Stevia* leaves are rich in PPs, flavonoids, and tannins, which exhibit strong antioxidant and antimicrobial activities. Terpenes and phenolic acids further enhance its medicinal value. These phytochemicals play critical roles in reducing oxidative stress, regulating blood sugar levels, and supporting cardiovascular health, making *Stevia* not only a natural sweetener but also a functional food ingredient.

*Stevia* contains numerous bioactive compounds, such as stevioside, rebaudioside (A, B, C, D, and E), steviolbioside, and dulcoside A, which are sweet diterpene glycosides [43]. Other phytochemicals identified in *Stevia* include austroinullin, β-carotene, dulcoside, niacin, rebaudiosides, riboflavin, steviol, stevioside, thiamine, caffeic acid, chlorogenic acid, trans-ferulic acid, and rutin [104]. *Stevia* is also a good source of vitamins, minerals, essential amino acids, fatty acids, and other beneficial bioactive compounds, including nonglycosidic diterpenes, flavonoids, phenolic compounds, crude fiber, phytosterols, chlorogenic acids, triterpenes, and hydrocarbons [105]. Furthermore, Milani et al. [106] identified stevioside (5%–10%), dulcoside (0.4%–0.7%), and rebaudioside (1%–2%) as key components in *Stevia* leaves. The plant also contains alkaloids, flavonoids, lutein, chlorophyll, hydroxycyanate, oligosaccharides, amino acids, free sugars, and other bioactive segments in its dry leaves [67].

Phytochemicals are plant-derived compounds produced through primary or secondary metabolism [107]. They exhibit biological activity in the host plant, playing roles in growth, defense against competitors, pathogens, or predators [108]. These compounds possess strong antioxidant properties, helping to prevent cellular damage caused by free radicals [109]. *Stevia* contains a variety of phytochemicals, including chlorogenic acids, folic acid, austroinullin, stevioside, β-carotene, nonglycosidic diterpenes, steviol, thiamine, riboflavin, glucoside, niacin, anthraquinones, rebaudiosides, vitamin C, oxides, and flavonoids, which contribute to its antioxidant and health-promoting properties [67]. *Stevia* is particularly rich in flavonoids and polyphenolic compounds, which exhibit strong antioxidant properties. These compounds help combat oxidative stress, potentially benefiting fish health by supporting the immune system and enhancing resistance to environmental stressors. Dry extracts of stevia leaves contain flavonoids, alkaloids, chlorophylls, xanthophylls, hydroxycinnamic acids (e.g., caffeic acid, chlorogenic acid), oligosaccharides, free sugars, amino acids, lipids, and trace elements [47]. Plant-derived active ingredients, such as phenolic compounds and flavonoids, can exert various effects on animals, including appetite stimulation, antimicrobial activity, immune system enhancement, and anti-inflammatory and antioxidant properties [110–112].

#### 2.3.1. Steviol Glycosides: Stevioside, Rebaudiosides (Main Sweetening Agents)

Steviol glycosides are the most biologically active components of *Stevia* leaves, accounting for their intense sweetness. These glycosides, including stevioside and rebaudiosides (particularly Reb A), represent a significant portion of the leaf's dry weight but have minimal caloric value. *Stevia* also contains proteins, fibers, and other phytochemicals. Researchers have identified several steviol glycosides, including stevioside, rebaudioside, dulcoside A, rebaudioside C, rebaudioside, diterpene glycosides, and steviolbioside [69, 113].

In 2017, the Food and Drug Administration (FDA) approved pure steviol glycosides for use in the food industry. The FDA classified different glycosides extracted from *Stevia* as steviosides, rebaudiosides, and dulcosides. *Stevia* leaves contain 7%–15% steviol glycosides, with stevioside (4.0%–8.5%), Reb A (1.5%–5%), rebaudioside C (0.1%–2.5%), and dulcoside A (0.1%–1%) being the primary sweetening compounds. However, advancements in plant breeding have led to higher steviol glycoside content (13%–20%) and increased Reb A levels (5%–12%) in newer varieties [24]. Stevioside and Reb A are the most prominent glycosides, contributing to the plant's intense sweetness. These compounds are zero-calorie, heat-stable, pH-stable, and resistant to fermentation [114], making them suitable for both human and animal nutrition. The extraction of stevioside and Reb A from *S. rebaudiana* leaves involves two stages: initial extraction of steviol glycosides using water, followed by purification through alcohol/water crystallization to yield highly pure compounds [115]. Modern extraction processes involve hot-water extraction of *Stevia* leaves, yielding a “primary extract” from which other plant components are removed via flocculation. The approximate dry weight percentages of various steviol glycosides present in *Stevia* leaves are as follows.

##### 2.3.1.1. Stevioside

Stevioside, first isolated by Bridel and Lavieille in 1931, is the most abundant glycoside in *Stevia* leaves, accounting for 5%–10% of its sweetness. However, the roots of the plant, which are fibrous and perennial, do not contain stevioside [116]. Stevioside is heat-stable up to 120°C, acid-stable, and nonfermentable [117]. Numerous studies have evaluated stevioside in both ruminant and nonruminant diets due to its stability under various conditions, noncaloric nature, and safety. Research has demonstrated that stevioside acts as a promising immunomodulator [118], exhibits bactericidal effects under acidic conditions [119], enhances feed intake [33], improves growth performance, boosts liver antioxidant capacity, and has immune-stimulating, physiological, and nutritional effects in aquatic animals [16].

Feed palatability, which includes physical qualities (e.g., appearance, texture) and chemical properties (e.g., taste, smell), is a critical factor influencing feed consumption in animals. Studies have shown that low concentrations of stevioside solutions (0.0003%–0.0005%) significantly influence the feeding behavior of yellow catfish [120]. Recent findings by Shehata et al. [21] indicate that adding stevioside at 450 mg/kg improves performance and health in *Liza ramada*. Similarly, common carp exhibited enhanced growth with *Stevia* extracts at 2000 mg/kg [121]. These variations may be attributed to species-specific attractant responses [16] and differences in digestive enzyme activities [122].

In *L. ramada* diets, stevioside may promote growth by enhancing feed palatability [21], resistance to digestive enzymes, and modulation of gut microbiota [14]. It also provides protective, bactericidal, and anti-inflammatory benefits, potentially leading to improved immune function and growth performance [33, 123]. Certain fish feed ingredients, such as fishmeal, can have elevated histamine levels, especially if subjected to excessive heat processing or spoilage. Heat treatment during fishmeal production can trigger a reaction between histidine and proteins, leading to histamine formation [124]. Histamine, a bioactive monoamine produced through the decarboxylation of L-histidine by histidine decarboxylase [34], has been linked to reduced intestinal immunity, diminished antioxidant defenses, oxidative damage in the digestive system, and impaired growth in species such as striped catfish [125]. In a study by Watanabe et al. [126], rainbow trout fed diets containing 0.1% histamine exhibited severe gastric damage, including erosion of 80% of the inner stomach lining, necrosis of gastric gland cells, and a significant reduction in stomach wall thickness. These findings highlight the detrimental effects of excessive histamine on fish health, particularly affecting the gastrointestinal system.

*Stevia* has demonstrated protective effects against histamine toxicity in both fish and terrestrial animals [34, 127]. *Stevia* extract helped rainbow trout (*O. mykiss*) avoid intestinal damage caused by histamine and maintained stable liver tocopherol levels. Researchers attribute these protective effects to *Stevia*'s antioxidant properties, its ability to inhibit histamine absorption in the intestine, and its suppression of excessive peptic activity that may irritate the digestive tract. *Stevia* may also inhibit H+/K+-ATPase activity, preventing significant pH changes and facilitating the breakdown of histamine into less harmful compounds [34]. Conversely, Choy et al. [128] suggest that *Stevia*'s protective effects against histamine may be less effective in tilapia due to their reduced sensitivity to histamine. The naturally low stomach pH of tilapia, which helps reduce histamine toxicity, may explain this reduced sensitivity [129]. Consequently, the incorporation of *Stevia* into tilapia diets may not provide significant benefits in terms of mitigating histamine-induced harm.

##### 2.3.1.2. Dulcoside A

Dulcoside A, with the molecular formula C~38~H~60~O~17~, has a melting point of 193–195°C and a solubility of 0.58 in water at 25°C [115]. *Stevia* leaves contain this compound in smaller quantities (0.4%–0.7%) [130]. Dulcoside A is one of the naturally occurring steviol glycosides found in *S. rebaudiana*. Structurally, dulcoside A consists of a steviol backbone linked to two glucose molecules, one at the C13 position and another at the C19 position. This configuration is similar to other steviol glycosides like stevioside and Reb A, but the number and arrangement of glucose molecules influence its sweetness and taste profile. Research indicates that dulcoside A is less sweet than stevioside and Reb A, contributing to the overall sweetness of *Stevia*, but to a lesser extent [117, 131]. Currently, there is limited research on the specific role of dulcoside A in fish nutrition. However, studies on the use of steviol glycosides, including those present in *Stevia* extracts, suggest several potential benefits in aquaculture. These benefits include: (1) improved palatability, potentially leading to increased feed intake; (2) antioxidant properties that could reduce oxidative stress in fish, promoting better health and growth [10]; and (3) immune modulation, potentially enhancing the immune response in fish [12]. Given these attributes, dulcoside A could be further explored as a natural additive in fish feed formulations, particularly for its potential health benefits.

##### 2.3.1.3. Reb A

Reb A, with the molecular formula C~44~H~70~O~23~, has a melting point of 242–244°C and a solubility of 0.80 in water at 25°C [115]. Reb A, also known as rebiana, is one of the most abundant and sweetest steviol glycosides found in *S. rebaudiana*. It consists of a steviol backbone linked to four glucose molecules, two at the C13 position and two at the C19 position. This configuration gives Reb A its distinct, intense sweetness, which is ~200–400 times sweeter than sucrose, with minimal bitterness compared to other steviol glycosides like stevioside [117]. Reb A and stevioside are the primary steviol glycosides in *S. rebaudiana* and are the two principal derivatives used in high-potency sweeteners. The absence of one glucose moiety distinguishes stevioside from Reb A. Food products commonly use rebaudioside A (2%–4%) due to its pleasant taste. In the gut, intestinal microflora (e.g., *Bacteroides* spp.) metabolize both Reb A and stevioside using the same mechanisms as steviol in rats, humans, and other species [132, 133].

The use of Reb A and *Stevia* extracts in aquaculture is relatively recent, with studies exploring their benefits beyond sweetness, particularly in enhancing fish health and growth. Some potential roles of Reb A in fish nutrition include: (1) enhancing feed palatability, leading to increased feed intake, especially in feeds containing less palatable but nutritious ingredients [10]; (2) possessing antioxidant properties that reduce oxidative stress in fish, thereby enhancing health, reducing disease risk, and potentially accelerating growth rates [131, 134]; (3) acting as an immunostimulant, helping fish combat infections and stress factors, particularly in intensive aquaculture systems [128]; and (4) serving as a potential growth promoter [33]. Further research is necessary to fully understand the potential benefits of Reb A in fish diets, particularly as a natural additive that can improve palatability, antioxidant defense, and immune function, and optimize its use in aquaculture nutrition.

##### 2.3.1.4. Rebaudiosides B, C (1%–2%), D, E, F, and M

Rebaudiosides B, C, D, E, F, and M are among the steviol glycosides found in *S. rebaudiana* leaves, although they are present in much lower concentrations compared to Reb A. Rebaudioside B (C_38_H_60_O_18_) is structurally similar to stevioside, with additional glucose molecules. Rebaudioside B has a moderate sweetness level, which is less than that of Reb A, and its lower abundance makes it less commonly extracted for commercial use. Rebaudioside C (C_44_H_70_O_22_) is known for its moderate sweetness and slightly bitter aftertaste. It comprises around 0.6% of the total steviol glycosides in *Stevia* leaves. Its limited sweetness and slight bitterness reduce its desirability as a standalone sweetener, but it may contribute to the overall sweet profile of *Stevia* extracts. Rebaudioside D (C_50_H_80_O_28_) is sweeter than both Reb A and C and is gaining attention due to its clean taste profile with minimal bitterness. It is ~200–300 times sweeter than sucrose, making it a desirable alternative in reduced-calorie food and beverage formulations. Rebaudioside D (C_50_H_80_O_28_) is sweeter than both Reb A and C and is gaining attention due to its clean taste profile with minimal bitterness. It is about 200–300 times sweeter than sucrose, making it a desirable alternative in reduced-calorie food and beverage formulations. Rebaudioside E (C_44_H_70_O_23_) has a similar sweetness intensity to Rebaudioside D but is less common in commercial products due to its limited availability in the *Stevia* plant. Researchers are exploring its potential synergistic effects with other sweeteners. Rebaudioside F (C_43_H_68_O_22_) is among the less studied steviol glycosides and is present in trace amounts in *Stevia* leaves. It has a sweetness profile similar to that of other minor glycosides, but its role in flavor enhancement is still under research. Rebaudioside M (C_56_H_90_O_33_) is one of the newer glycosides to be commercialized, recognized for its highly desirable sweetness profile. It is about 200 times sweeter than sucrose and has a clean taste with minimal bitterness, which makes it particularly suitable for sugar replacement. Rebaudioside M has the potential for broader use in the food industry, as it can achieve higher sweetness levels without the aftertaste associated with other *Stevia* glycosides. The exploration of these compounds is ongoing, especially regarding their sensory properties, metabolic effects, and potential health benefits [113, 135].

##### 2.3.1.5. Rubusoside and Steviolbioside

These are present in trace amounts, with rebaudioside B sometimes resulting from isolation techniques [27]. Rubusoside (C_32_H_50_O_13_) consists of a steviol backbone linked to two glucose molecules in a specific configuration. Conversely, steviolbioside (C_26_H_36_O_13_) combines a steviol aglycone with one glucose molecule and one rhamnose molecule. It is also a steviol glycoside, albeit less sweet compared to others like Reb A [136]. Approximately 80% stevioside, 8% Reb A, and 0.6% rebaudioside C typically make up a commercial steviol glycoside extract [137]. Some studies have highlighted the potential benefits of stevioside in aquaculture, demonstrating antioxidant and immunomodulatory properties across various biological systems [16, 138]. Additionally, stevioside exhibits attractant properties akin to saccharides and sugar alcohols in fish and shellfish [21].

Sweet substances favored by humans are also attractive to a wide range of aquatic species, including teleosts, echinoderms, crustaceans, mollusks, coelenterates, and protozoans [139]. Aquatic animals naturally encounter saccharides, sugar alcohols, and amino acids, but their diets rarely contain certain herbal compounds with high concentrations of sweet substances [21]. Although specific behavioral responses to stevioside in fish and crustaceans remain insufficiently documented, some studies suggest it may enhance feed palatability and voluntary feed intake, indicating a potential role as a feeding stimulant [21, 22]. However, further research is needed to confirm species-specific behavioral effects, such as feeding motivation or preference. Although stevioside is generally considered safe for human consumption, its effects on aquatic organisms have piqued scientific interest [41]. In an effort to increase profitability and sustainability, the aquaculture sector has explored using by-products like stevioside in fish diets. Research has shown that stevioside can act as an immunostimulant, enhancing immune responses in fish [12].

Young black abalone (*H. discus*), adult oriental weather fish (*Misgurnus anguillicaudatus*), and juvenile yellowtail (*Seriola quinqueradiata*) were all attracted to *Stevia* in early tests [140]. Stevioside is primarily responsible for the attractive effect. While saccharides and sugar alcohols have well-documented attractant capabilities, there is less research on similar properties for glycosides like stevioside, which suggests that they could be potential attractants in aquaculture [139]. Incorporating these glycosides into aquafeeds could improve feed palatability and intake in fish [33, 141]. Choy et al. [128] examined the effects of adding different amounts of *Stevia* (0%, 1%, 3%, and 6%) to tilapia diets. They found that the levels of *Stevia* did not have a significant effect on the fish's ability to taste, eat, or grow. This implies that the fish may not benefit from these levels of *Stevia* in their diet. The authors suggested that *Stevia* may not be metabolized as an energy source in tilapia.

Also, Shiozaki et al. [34] found that the addition of *Stevia* leaf extract at 0.2% had no effect on the growth, feeding efficiencies, or body indices of rainbow trout (*O. mykiss*). Certain ingredients, like fishmeal, have a high histamine content, especially when they undergo excessive heat processing or begin to deteriorate [124]. *Stevia* is known to have some protective effects against histamine toxicity in fish's stomachs, which may be attributed to its antioxidant properties: blocking histamine intestinal absorption, preventing excessive peptic activity that would irritate the intestine, inhibiting H/K-ATPase activity, pH changes, and reducing histamine into less toxic components [34]. Han et al. [33] examined different doses of stevioside in goat diets and found that *Stevia* inclusion levels commonly used in fish feed were too high. Aquafeeds generally incorporate sweeteners at low concentrations, typically around 0.05%–0.2% of total feed. For instance, Wang et al. [16] looked at *Stevia* levels in *Cyprinus carpio* diets that ranged from 0 to 400 mg/kg and found that 217.68 mg/kg (0.022 g/100 g) was the best dose for improving growth, liver antioxidant capacity, and immune function.

### 2.4. PPs: Antioxidants Like Flavonoids and Phenolic Acids

#### 2.4.1. Phenolic Acids

Researchers discovered polyphenolic compounds with antioxidant properties in the leaves of *S. rebaudiana* Bertoni [142–144]. Phenolic acids or phenolcarboxylic acids are phenolic compounds and types of aromatic acid compounds. Included in that class are substances containing a phenolic ring and an organic carboxylic acid function (C_6_–C_1_ skeleton) [145]. Plant phenols are a large and diverse group of compounds, including hydroxycinnamates, tannins, flavonoids, stilbenes, coumarins, lignans, and lignins [146]. The most common hydroxycinnamate derivatives found in plants are chlorogenic acids, such as caffeic acid (C_9_H_8_O_4_) and chlorogenic acid (C_16_H_18_O_9_), both of which fight free radicals. In *Stevia* leaves, the mass content of PPs is around 2%–5% of dried leaves. Researchers have reported finding more than 30 *Stevia* phenolic compounds from leaves, in addition to 64 steviol glycosides [147–150]. PPs in *Stevia* might affect its organoleptic properties and inhibit hydroperoxide formation in fish oil [149]. The beneficial effects of PPs have been attributed to their strong antioxidant activity, neutralizing free radicals, and anti-inflammatory and antimicrobial properties [151]. Total phenolic content of *S. rebaudiana* was achieved, containing 61.50 mg/g [143].

Aquaculture feed additives boost fish growth, immunology, and antioxidant status and prevent diseases. Among these feed additives, phytochemicals draw the most attention. Phytochemicals are considered harmless for humans, fish, and the environment because they are natural products. Ferulic acid is a phenolic substance with numerous advantages linked to its antioxidant, cytoprotective, antibacterial, and anabolic activities, generating particular interest in aquaculture. The stimulation and favoring of these additives lead to the stimulation of physiological and metabolic processes, promoting aquatic animals' development and reproduction. Therefore, the administration of ferulic acid could enhance the growth of muscle fibers, immunological response, inflammatory response, and antioxidant capacity in fish [152]. Phenolic chemicals are among the many natural products found in marine green algae belonging to the genus Ulva [153].

#### 2.4.2. Flavonoids

Flavonoids, also known as bioflavonoids, are polyphenolic secondary metabolites widely distributed in plants and commonly consumed in the human diet [154]. Based on their chemical structure, we classify them into six primary classes: flavonols, flavones, isoflavones, flavan-3-ols (catechins), flavanones, and anthocyanins, which exhibit structural diversity. Each class consists of specific compounds with unique functional properties. The main classes and subclasses of flavonoids include: (1) flavonols, such as quercetin, kaempferol, and myricetin, which are found in fruits, vegetables, and tea, and are known for their antioxidant properties. (2) Flavones, which include apigenin and luteolin, found in parsley, celery, and citrus fruits, exhibit anti-inflammatory and antioxidant effects. (3) Soy and legumes predominantly contain isoflavones, such as genistein and daidzein, which are known for their estrogen-like activity. (4) Flavan-3-ols (catechins) include catechin, epicatechin, and epigallocatechin gallate, found in tea, cocoa, and berries; they have strong antioxidant properties. (5) Flavonoids, including naringenin and hesperidin, are abundant in citrus fruits and contribute to bitter flavor and antioxidant activity. (6) Berries, grapes, and red cabbage contain anthocyanins, such as cyanidin, delphinidin, and malvidin, which are responsible for the red, blue, and purple pigmentation [155, 156]. Flavonoids protect plants against UV radiation and pathogens, while in humans and animals, they provide health benefits such as antioxidation, anti-inflammation, and enhanced immunity [157].

Chemically, flavonoids share a common 15-carbon skeleton with two phenyl rings (A and B) and a heterocyclic oxygen-containing ring (C) [154, 158]. Among flavonoids, quercetin, kaempferol, and rutin may stand out for their roles in enhancing fish health. These compounds improve oxidative stress responses, modulate inflammatory pathways, and boost immunity. For example, the structure of kaempferol and quercetin, two important flavonols, is different because quercetin has an extra hydroxyl group that affects its biological activities. Additionally, flavonoids contribute to food color, flavor, and protection against nutrient degradation, such as lipid oxidation [155, 157]. Chalcones, aurones, flavonols, anthocyanins, proanthocyanidins, and condensed tannins are subclassifications of flavonoids that reflect their structural complexity and diverse biological roles [156].

##### 2.4.2.1. Quercetin

Quercetin is a polyphenolic flavonol widely found in fruits, vegetables, and plants such as onions, apples, broccoli, tea, and Stevia [159, 160]. Structurally, it is characterized by a hydroxyl-rich aromatic ring system, including an extra hydroxyl group at the third position of its B-ring. This structure underpins its strong antioxidant, anti-inflammatory, and immunomodulatory properties, making quercetin one of the most extensively studied flavonoids [161, 162]. In aquaculture, quercetin may support oxidative stress management by scavenging free radicals and enhancing antioxidant enzyme activity, such as superoxide dismutase (SOD) and catalase (CAT). This aids in preserving cellular integrity and mitigates tissue damage resulting from metabolic or environmental stress. Furthermore, quercetin may enhance fish immunity by modulating inflammatory pathways and boosting disease resistance, leading to improved survival rates and stress tolerance [163]. Quercetin's antiviral and antimicrobial activities may also contribute to fish health, positioning it as a promising natural alternative to synthetic additives in sustainable aquaculture. Its ability to reduce metabolic stress and improve nutrient utilization further highlights its role in supporting growth and overall fish welfare [164].

##### 2.4.2.2. Kaempferol

*S. rebaudiana* contains kaempferol, a widely distributed flavonol, as one of its phenolic compounds. This natural antioxidant contributes to the plant's ability to combat oxidative stress and its health-promoting effects when consumed. Kaempferol is primarily known for its role in scavenging free radicals and inhibiting lipid peroxidation, which helps maintain cellular integrity and reduce oxidative damage [165]. In *Stevia*, kaempferol is often associated with other phenolics, such as quercetin, enhancing the plant's overall antioxidant and anti-inflammatory properties. These compounds work synergistically to support health benefits, including improved immune modulation. Studies have also indicated kaempferol's potential role in inhibiting the growth of certain pathogens, lowering the risk of infections, adding to its significance as a component of *Stevia* in promoting both plant resilience and consumer health [67, 166]. However, reports suggest that a kaempferol overdose may cause self-oxidation in humans but has no effect on animals [167].

##### 2.4.2.3. Rutin

Plants, including *S. rebaudiana*, widely contain rutin, a glycosylated derivative of quercetin, a bioactive flavonoid. It shares similar bioactivities with quercetin, such as potent antioxidant and anti-inflammatory effects [166]. In plants, rutin acts as a secondary metabolite, providing protection against oxidative stress and UV damage, contributing to the plant's resilience and medicinal properties. In aquaculture, rutin may have demonstrated significant potential as a functional feed additive. It enhances antioxidant enzyme activities, such as SOD and CAT, which are essential for mitigating oxidative stress and maintaining physiological stability under challenging conditions. These effects can improve survival rates, enhance disease resistance, and improve growth performance in fish. The inclusion of rutin in aquatic feeds exemplifies its role in sustainable aquaculture practices by supporting fish health and resilience to environmental stressors [165].

### 2.5. Tannins: Bioactive Plant Compounds

Tannins, a class of polyphenolic compounds, are bioactive components present in smaller amounts in *S. rebaudiana*. Manish Tadhani and Rema Subhash [44] found that the most abundant compounds in *S. rebaudiana* leaf extract were tannins and alkaloids. These phenolic metabolites are characterized by their ability to strongly complex with carbohydrates and proteins [168]. The two main types of tannins are proanthocyanidins (condensed tannins) and hydrolyzable tannins, which differ in their chemical structures and biological properties [169]. Additionally, the solubility of tannins in water distinguishes soluble and insoluble tannins [170]. Tannins serve as defense compounds in plants, protecting them against herbivores, pathogens, and oxidative stress. Their antioxidant properties are well-documented, with tannins efficiently scavenging free radicals and shielding cellular components from oxidative damage [171]. They also exhibit antimicrobial and anti-inflammatory activities. In aquaculture, tannins may have benefits in improving gut health, modulating gut microbiota, and enhancing immune responses in fish. These effects promote better nutrient absorption, stress resistance, and growth performance [67]. Phlorotannins, a type of specialized tannin present in marine brown algae, demonstrate distinct bioactivities, thereby broadening the role of tannins in aquatic ecosystems [172]. From their chemical structure, tannins may also be divided into soluble and insoluble tannins [170]. Tannins mainly affect living things by binding to metal ions, being an antioxidant, scavenging radicals, and forming complexes with proteins and polysaccharides [173]. The presence of tannins in *Stevia* supports its potential as a functional ingredient in aquatic feeds, aligning with sustainable and health-oriented aquaculture practices.

### 2.6. Terpenes

Terpenes and essential oils are significant bioactive components found in *S. rebaudiana*. Terpenes, a large class of natural compounds composed of isoprene units, play critical roles in plant defense against herbivores and pathogens, as well as in attracting pollinators through their aromatic properties [174]. In *Stevia*, terpenes contribute to its characteristic aroma and biological activity, including antioxidant, anti-inflammatory, and antimicrobial properties [67]. Essential oils, which are concentrated, water-repellent liquids containing volatile terpene derivatives, exhibit strong antimicrobial and antioxidant properties, further enhancing the medicinal value of *Stevia*. Terpenes such as limonene, linalool, and caryophyllene are present in *Stevia* and are associated with its health-promoting properties, including its ability to combat oxidative stress and inhibit microbial growth [175]. These compounds may also modulate metabolic pathways in animals, enhancing the overall physiological resilience of aquaculture species. The integration of terpene-rich components like *Stevia* into fish diets offers potential benefits for improving growth performance, immunity, and stress tolerance, promoting sustainable and health-oriented aquaculture practices.

## 3. Functional Properties of *Stevia* Leaves

*Stevia* leaves exhibit unique functional properties that make them suitable for various applications in the food and feed industries.

### 3.1. Phenolic Compounds

The phenolic compounds found in *Stevia* leaves include chlorogenic acids, members of the polyphenol family of esters, and hydroxycinnamic acid esters, which exhibit excellent hydrophilic antioxidant activity and therapeutic properties [151]. The glycosides in *Stevia* leaves are non-nutritive sugar substitutes, meaning they do not provide energy upon consumption. The literature highlights the antioxidant properties and sweet diterpenes of *Stevia* leaves, in addition to their sweetening properties [147]. *S. rebaudiana*, a chrysanthemum herb, is used as a vegetable-based sweetening additive in health drinks and various food products. Studies have investigated the antioxidant activity and presence of bioactive compounds in *Stevia* leaves. Analysis shows that *Stevia* leaves contain folic acid at 52.18 mg/100 g. Additionally, the leaves contain 130.76 µg of catechin and 15.64 µg of quercetin, while water extracts contain 43.99 µg of catechin and 1.57 µg of quercetin. Pyrogallol is the major phenolic compound present in both leaf and cellular extracts of *Stevia*. Several studies have also shown that leaf extracts exhibit higher free radical, hydroxyl radical, and superoxide anion radical scavenging activities compared to cellular extracts [176].

### 3.2. Glycoside Compounds

A glycosidic bond connects a sugar group to another functional group in a glycoside. In living organisms, glycosides perform vital roles. Numerous plant cells store glycosides in an inactive form [177]. The sweet leaves of *Stevia* contain glycosides such as stevioside, steviol, steviolbioside, Reb A, rebaudioside B, rebaudioside C, rebaudioside D, and dulcoside A [178].

### 3.3. Effects of *S. rebaudiana* on Performance in Animal and Aquaculture

The use of *S. rebaudiana* as a feed additive in animal husbandry and aquaculture has attracted considerable interest due to its potential to improve multiple facets of performance and health. This section examines the diverse effects of *S. rebaudiana* on essential parameters, as outlined in Table 3. The themes addressed include growth performance, lipid metabolism, antioxidant activities, and immunological responses. *S. rebaudiana*, a naturally occurring substance derived from a variety of plant sources, has shown promise as a bioactive supplement that can benefit aquatic and animal species. It works through various mechanisms, from cellular-level physiological process modulation to systemic effects that result in enhanced performance. We will explore the complex interactions between *S. rebaudiana* and the selected performance metrics in this section. Our goal is to provide a comprehensive understanding of the potential benefits that supplementing *S. rebaudiana* may offer to the aquaculture and animal husbandry industries. This investigation will enhance our understanding of the physiological mechanisms underlying the observed effects, thereby facilitating informed decision-making regarding the optimal management of animal nutrition and health.

### 3.4. Growth Performance

Stevioside is considered a safe substance. The study examined the effects of supplementing juvenile thinlip mullet (*L. ramada*) with stevioside and lead on their growth performance and feed efficiency. This study indicated that the supplementation of stevioside at different concentrations (150, 250, 350, and 450 mg/kg), along with a constant lead (Pb) concentration of 100 μg/kg, positively affected growth parameters, with the 450 mg/kg + Pb treatment yielding the highest values [21]. In addition, Wang et al. [16] discovered that *Stevia* extract at a dose of 217.68 mg/kg improved the growth of young mirror carp (*C. carpio*) and made them gain weight at the same rate as the control group. Wang et al. [141] indicate that dietary supplementation with stevioside/rebaudioside enhances growth performance and decreases the incidence of diarrhea in weaned pigs. The ideal dosage for food stevioside supplementation in weaned pigs' diets was 250 mg/kg; the ideal dosage for dietary rebaudioside supplementation in the feed of weaned pigs ranged from 191 to 213 mg/kg. Additionally, the study examined the impact of dietary stevioside levels on the growth performance of broiler chicks during the finisher stage (19–35 days). The results demonstrated that, in comparison to the control group, stevioside supplementation significantly increased body weight gain and feed consumption at 19–35 days [180]. Researchers conducted a previous study to explore the effects of stevioside on broilers. In comparison to the other three groups, the broilers that were fed a diet consisting of steviosides had higher body weights when they were 4 weeks old [179].

### 3.5. Antioxidants Activities

Antioxidant activity is critical for maintaining cellular health and preventing oxidative damage. The study examined the impact of varying levels of stevioside supplementation (0, 150, 250, 350, and 450 mg/kg) on the antioxidant enzyme activities of CAT, glutathione peroxidase (GPx), and SOD in *L. ramada*, which had consumed a high-lead diet. The results show that being exposed to lead greatly lowered the activities of SOD, CAT, and GPx when compared to a healthy control group that did not receive any lead. However, coadministration of stevioside with lead produced a dose-dependent protective effect. The groups that were given 250, 350, and 450 mg/kg of stevioside had the same levels of SOD, CAT, and GPx activity as the healthy control group [21, 22]. A recent study also found that steviosides responded quadratically to the SOD activity of *C. carpio*. Stevioside supplementation did not influence malonaldehyde (MDA) content or CAT activity. The best amount of steviosides to give was 215.21 mg/kg, based on the broken-line regression model used to measure SOD activity [16]. In 2024, Wen et al. [179] showed that adding stevia extract to the liver increased its total antioxidant capacity and total SOD by the end of the 24th week. Stevia extract increased serum total SOD in laying hens in a linear manner, as indicated by serum antioxidant levels [179].

### 3.6. Immune Responses and Lipid Metabolism

Stevioside and Rebaudioside, derived from *S. rebaudiana*, are essential for immunological regulation and lipid metabolism in animal husbandry and aquaculture. Their capacity to enhance immunity, diminish inflammation, and optimize lipid utilization renders them valuable as natural feed additives. In line with sustainable and health-focused farming methods, these compounds also improve the health and quality of aquatic and animal species. In aquaculture, Shehata et al. [21] reported that stevioside (450 mg/kg diet) improved biochemical parameters, immune responses, and the expression of *IL-1β* and *hepcidin* genes in *L. ramada* exposed to lead, indicating a protective role against heavy metal toxicity. Similarly, supplementation in mirror carp enhanced immunological responses without affecting crude lipid levels [16]. In poultry, the inclusion of 200 mg/kg stevia extract in laying hen diets improved production performance, immune function, and gut health, though no significant effects were observed on liver function or lipid metabolism [179]. Stevioside also increased total protein, globulin, immunoglobulin A (IgA), and immunoglobulin G (IgG) concentrations in broilers, reflecting enhanced immune status [180]. While it did not alter the fatty acid profile, hens exhibited improved physiological responses [181]. In ruminants, Reb A supplementation at 350–700 mg/kg in adult goats improved feed intake and metabolic efficiency [182]. Additionally, dietary inclusion of stevioside at 400–800 mg/kg enhanced the digestibility of neutral and acid detergent fiber across the digestive tract [33]. In rodent models, low-dose stevioside administration (15 mg/kg/day) in young growing rats for 12 weeks improved hematological and biochemical indices [183]. Furthermore, the combination of stevioside with soy protein isolate was shown to reduce total cholesterol and alleviate symptoms of metabolic syndrome in Goto-Kakizaki rats [185].

## 4. Conclusion

*S. rebaudiana* demonstrates considerable potential as a natural additive in animal feed and aquaculture diets. Its bioactive components, particularly stevioside and Reb A, contribute to enhanced immune response, improved lipid metabolism, and increased resistance to oxidative stress. These effects collectively improve overall health, reduce reliance on synthetic antibiotics, and enhance the quality of animal husbandry and aquaculture products. *S. rebaudiana*, by advocating sustainable agricultural methods, presents a viable substitute for traditional feed additives. Its application corresponds with the worldwide demand for healthier animal products and sustainable agricultural practices. Future evaluations should also include economic indicators such as production costs, scalability, and market potential, as well as environmental metrics, including water usage, carbon footprint, and effects on soil health, to support holistic sustainability assessments.

## 5. Future Prospects

The incorporation of *S. rebaudiana* in animal feed and aquaculture diets presents multiple avenues for additional research and development as follows:

1. Additional studies are necessary to establish species-specific optimal dosages of *S. rebaudiana* to maximize health and productivity outcomes.
2. Research into the formulation of Stevia-based feeds is essential to ensure scalability, formulation stability, and cost-effectiveness in commercial applications.
3. It is important to look into the exact molecular pathways that stevioside and Reb A use to affect metabolic and immune functions. Future research should particularly focus on signaling pathways such as NF-*κ*B, Nrf2/Keap1, and MAPK, as well as key immune and antioxidant-related target genes like *IL-1β*, *TNF-α*, *SOD*, *CAT*, and *GPx*.
4. There is a need to evaluate the long-term effects of *S. rebaudiana* supplementation on animal health, fertility, and product quality.
5. A comprehensive assessment of the economic and environmental feasibility of large-scale *S. rebaudiana* cultivation and utilization within global agricultural systems is warranted.

By focusing on these aspects, *S. rebaudiana* could establish itself as a significant contributor to sustainable animal husbandry and aquaculture techniques.

## Figures and Tables

**Table 1:** Nutritional composition of *Stevia* leaves (g/100 g DW).

Nutritional components (g/100 g DW)					Reference
Protein	Fat	Carbohydrates	Ash	Dietary fiber	
9.63	3.47	66.50	3.08	17.12	[40]
16.0	2.60	—	15.5	6.8	[39]
11.4	3.73	61.90	7.41	15.5	[41]
11.2	5.60	53.0	—	15.0	[42]
11.2	1.90	—	6.3	15.2	[43]
20.42	4.34	35.2	13.12	—	[44]
12.0	2.7	—	8.4	—	[45]
10.0	3.0	52.0	11.0	18.0	[46]
10.0	3.0	52.0	11.0	18.0	[19]

**Table 2:** Vitamins and minerals contents of *Stevia* leaves.

Vitamins		Minerals			
[71, 72]		[44]		[39]	[19]
Water soluble vitamin	%	Mineral	%	%	%
Vitamin C	14.97	Na	0.16	0.7	0.19
Vitamin B_2_	0.43	K	2.51	17.3	1.8
Vitamin B_6_	1.90	P	0.35	2.6	0.01
Folic acid	52.18	Mg	0.50	2.4	—
Niacin	194	Fe	0.0363	0.0366	0.06
Thiamine	18.8	Zn	0.00639	0.002	—

**Table 3:** *Stevia rebaudiana* enhances animal growth, lipid metabolism, antioxidant activity, and immunity.

No.	Animal species	Common names and abbreviations	Tested dosage	Duration	Route of administration	Performance effects (growth, lipid metabolism, antioxidant, and immunity^a^	References
1	Mullet (*Liza ramada*)	Stevioside (Stev)	0, 150, 250, 350, and 450 mg/kg diet	60 days	Diet	Positive impacts (↑)	[21, 22]
2	Carp (*Cyprinus carpio*)	Stevioside (STE)	0, 80, 160, 240, 320, and 400 mg/kg diet	56 days	Diet	Positive impacts (↑)	[16]
3	Laying hens	Stevia extract	0, 50, 100, 200, and 400 mg/kg diet	24 weeks	Diet	Positive impacts (↑)	[179]
4	Weaned piglets	Stevioside	0, 100, 150, 200, 250, and 300 mg/kg diet	42 days	Diet	Positive impacts (↑)	[141]
5	Broiler	Stevioside (STE)	0, 1.5, 3, and 6 g/kg diet	45 days	Diet	Positive impacts (↑)	[180]
6	Cobb broiler chicks	Stevia and stevioside	2% stevia leaves and 130 ppm stevioside	4 weeks	Diet	Positive impacts (↑)	[181]
7	Adult goats	Rebaudioside	0, 350, and 700 mg/kg rice straw (DM)	21 days	Diet	Positive impacts (↑)	[182]
8	Goats	Stevioside	0, 400, and 800 mg/kg rice straw (DM)	20 days	Diet	Positive impacts (↑)	[33]
9	Male rats	Stevioside (S)	Low dose: 15 mg/kg/day; high dose: 100 × low dose	12 weeks	Diet	Positive impacts (↑)	[183]
10	Broiler chickens	Stevioside (STE)	0 and 250 mg/kg diet	21 days	Diet	Positive impacts (↑)	[184]
11	Rats	Stevioside	0 and 0.03 g/kg BW/day	4 weeks	Diet	Positive impacts (↑)	[185]

^a^(↑) = Significant.

## Data Availability

This review article utilized publicly available and cited data sources, including scholarly articles, books, reports, and online repositories. No new data were generated as part of this review. All sources referenced in this article are appropriately cited in the references section.

## References

[1] Choudhari A. S., Mandave P. C., Deshpande M., Ranjekar P., Prakash O. (2020). Phytochemicals in Cancer Treatment: From Preclinical Studies to Clinical Practice. *Frontiers in Pharmacology*.

[2] Mani J. S., Johnson J. B., Steel J. C. (2020). Natural Product-Derived Phytochemicals as Potential Agents Against Coronaviruses: A Review. *Virus Research*.

[3] Terzi E., Kucukkosker B., Bilen S. (2021). A Novel Herbal Immunostimulant for Rainbow Trout (*Oncorhynchus mykiss*) Against *Yersinia ruckeri*. *Fish & Shellfish Immunology*.

[4] Macías-Cruz U., Vicente-Pérez R., López-Baca M. A. (2018). Effects of Dietary Ferulic Acid on Reproductive Function and Metabolism of Pre-Pubertal Hairbreed Ewes During the Anestrous Season. *Theriogenology*.

[5] Marquardt R. R., Li S. (2018). Antimicrobial Resistance in Livestock: Advances and Alternatives to Antibiotics. *Animal Frontiers*.

[6] Kothari D., Patel S., Kim S.-K. (2019). Probiotic Supplements Might not be Universally-Effective and Safe: A Review. *Biomedicine & Pharmacotherapy*.

[7] Hashem N. M., Gonzalez-Bulnes A., Simal-Gandara J. (2020). Polyphenols in Farm Animals: Source of Reproductive Gain or Waste?. *Antioxidants*.

[8] Onomu A. J., Okuthe G. E. (2024). The Role of Functional Feed Additives in Enhancing Aquaculture Sustainability. *Fishes*.

[9] Shehata A. I., Rasheed M., Rafiq H. (2024). Multi-Functional Application of Octacosanol as a Feed Additive in Animal and Aquaculture: A Review. *Journal of Animal Physiology and Animal Nutrition*.

[10] Ahmad J., Khan I., Blundell R., Azzopardi J., Mahomoodally M. F. (2020). *Stevia rebaudiana*, Bertoni.: An Updated Review of Its Health Benefits, Industrial Applications and Safety. *Trends in Food Science & Technology*.

[11] Sakai M. (1999). Current Research Status of Fish Immunostimulants. *Aquaculture*.

[12] Leaño E. M. (2007). Effects of Stevia Extract on Growth, Non-Specific Immune Response and Disease Resistance of Grass Prawn, *Penaeus monodon* (Fabricius), Juveniles. *Journal of the Taiwan Fisheries Society*.

[13] Yanbo W., Zirong X. (2006). Effect of Probiotics for Common Carp (*Cyprinus carpio*) Based on Growth Performance and Digestive Enzyme Activities. *Animal Feed Science and Technology*.

[14] Kiron V. (2012). Fish Immune System and Its Nutritional Modulation for Preventive Health Care. *Animal Feed Science and Technology*.

[15] Ortiz L. T., Rebolé A., Velasco S. (2013). Effects of Inulin and Fructooligosaccharides on Growth Performance, Body Chemical Composition and Intestinal Microbiota of Farmed Rainbow Trout (*Ncorhynchus mykiss*). *Aquaculture Nutrition*.

[16] Wang J., Li K., Wang L., Xu Q. (2021). Effects of Different Levels of Stevioside on Growth Performance, Digestive Enzyme Activity, Antioxidant Capacity and Gene Expression of Juvenile Mirror Carp (*Cyprinus carpio*). *Aquaculture*.

[17] Bertoni M. S. (1905). *Le Kaá Hê-é, Sa Nature et ses Properties, An cie*.

[18] Ibrahim I. A., Nasr M. I., Mohammedm B. R., El-Zefzafi M. M. (2008). Nutrient Factors Affecting in Vitro Cultivation of *Stevia rebaudiana*. *Sugar Tech*.

[19] Savita S. M., Sheela K., Sunanda S., Shankar A. G., Ramakrishna P. (2004). *Stevia rebaudiana—*A Functional Component for Food Industry. *Journal of Human Ecology*.

[20] Jawad M., Rizwan B., Jawad M., Khalid F., Ishaq A. (2022). A Nutraceutical and Therapeutic Potentials of, *Stevia Rebaudiana*, Bertoni: Nutraceutical and Therapeutic Potentials of Stevia. *Pakistan BioMedical Journal*.

[21] Shehata A. I., Shahin S. A., Elmaghraby A. M. (2024). Stevioside Mitigates Lead Toxicity in Thinlip Mullet Juveniles: Impacts on Growth, Metabolism, and Immune Function. *Aquatic Toxicology*.

[22] Shehata A. I., El Basuini M. F., Elmaghraby A. M. (2025). Dietary Prebiotic-Stevioside Modulates the Growth, Antioxidant Enzymes, and Immune Response in Thinlip Mullets (*Liza ramada*) Subjected to Chronic Cold Stress. *BMC Veterinary Research*.

[23] King R., Robinson H. (1987). The Genera of the Eupatorieae (Asteraceae). Monographs in Systematic Botany.

[24] Kinghorn A. D. (2001). *Stevia: the Genus Stevia*.

[25] Arora S. (2015). *Nutritional Evaluation and Glycaemic Response of Food Products Prepared Using Stevia Rebaudiana*.

[26] Katayama O., Sumida T., Hayashi H., Mitsuhashi H. (1976). *The Practical Application of Stevia and Research and Development Data*.

[27] Brandle J. E., Starratt A. N., Gijzen M. (1998). *Stevia rebaudiana*: Its Agricultural, Biological, and Chemical Properties. *Canadian Journal of Plant Science*.

[28] Jahangir Chughtai M. F., Pasha I., Zahoor T. (2020). Nutritional and Therapeutic Perspectives of *Stevia rebaudiana* as Emerging Sweetener; a Way Forward for Sweetener Industry. *CyTA - Journal of Food*.

[29] Khalid W., Rehman A., Md Irfan Jha R., Md Khalid J., Aziz A. (2021). Nutritional Composition and Therapeutic Benefits of Stevia Leaves: A Mini Review. *Acta Scientific Microbiology*.

[30] Hossain M., Islam M., Islam M., Akhtar S. (2017). Cultivation and Uses of Stevia (*Stevia rebaudiana* Bertoni): A Review. *African Journal of Food, Agriculture, Nutrition and Development*.

[31] McMeniman J. P., Rivera J. D., Schlegel P., Rounds W., Galyean M. L. (2006). Effects of an Artificial Sweetener on Health, Performance, and Dietary Preference of Feedlot Cattle. *Journal of Animal Science*.

[32] Ponce C. H., Brown M. S., Silva J. S., Schlegel P., Rounds W., Hallford D. M. (2014). Effects of a Dietary Sweetener on Growth Performance and Health of Stressed Beef Calves and on Diet Digestibility and Plasma and Urinary Metabolite Concentrations of Healthy Calves. *Journal of Animal Science*.

[33] Han X., Chen C., Zhang X. (2019). Effects of Dietary Stevioside Supplementation on Feed Intake, Digestion, Ruminal Fermentation, and Blood Metabolites of Goats. *Animals*.

[34] Shiozaki K., Nakano T., Yamaguchi T., Sato M., Sato N. (2004). The Protective Effect of Stevia Extract on the Gastric Mucosa of Rainbow Trout *Oncorhynchus mykiss* (Walbaum) Fed Dietary Histamine. *Aquaculture Research*.

[35] Ramesh K., Singh V., Megeji N. W. (2006). Cultivation of Stevia [*Stevia rebaudiana* (Bert.) Bertoni]: A Comprehensive Review. *Advances in Agronomy*.

[36] Chatsudthipong V., Muanprasat C. (2009). Stevioside and Related Compounds: Therapeutic Benefits Beyond Sweetness. *Pharmacology & Therapeutics*.

[37] Takahashi K., Akiba Y., Nakano T., Yamaguchi T., Sato M., Sato N. (2001). Effect of Dietary Stevia (*Stevia rebaudiana*) Extract on Gizzard Erosion and UIceration Induced by Dietary Histamine in Broiler Chicks. *The Journal of Poultry Science*.

[38] Seidavi A., Hosseintabar-Ghasemabad B., Gorlov I. F., Arsenos G., Giannenas I. (2023). Feed Additives and Future Perspectives. *Sustainable Use of Feed Additives in Livestock: Novel Ways for Animal Production*.

[39] Atteh J., Onagbesan O., Tona K., Buyse J., Decuypere E., Geuns J. (2011). Potential use of *Stevia rebaudiana* in Animal Feeds. *Archivos de Zootecnia*.

[40] Elhassaneen Y. A. (2019). Stevia (*Stevia rebaudiana*) Leaves: Chemical Composition, Bioactive Compounds, Antioxidant Activities, Antihyperglycemic and Antiatherogenic Effects. *Journal of Studies and Searches of Specific Education*.

[41] Abou-Arab A. E., Abou-Arab A. A., Abu-Salem M. F. (2010). Physico-Chemical Assessment of Natural Sweeteners Steviosides Produced From, *Stevia rebaudiana*, Bertoni Plant. *African Journal of Food Science*.

[42] Serio L. (2010). La *Stevia rebaudiana*, Une Alternative au Sucre. *Phytothérapie*.

[43] Goyal S. K., Samsher, Goyal R. K. (2010). Stevia (*Stevia rebaudiana*) a Bio-Sweetener: A Review. *International Journal of Food Sciences and Nutrition*.

[44] Manish Tadhani M. T., Rema Subhash R. S. (2006). Preliminary Studies on *Stevia rebaudiana* Leaves: Proximal Composition, Mineral Analysis and Phytochemical Screening. *The Journal of Medical Sciences*.

[45] Kaushik R., Narayanan P., Vasudevan V., Muthukumaran G., Usha A. (2010). Nutrient Composition of Cultivated Stevia Leaves and the Influence of Polyphenols and Plant Pigments on Sensory and Antioxidant Properties of Leaf Extracts. *Journal of Food Science and Technology*.

[46] Mishra P. K., Singh R., Kumar U., Prakas V. (2010). *Stevia rebaudiana*–A Magical Sweetener. *Global Journal of Biotecnology & Biochemistry*.

[47] Silva G. E. C. d., Assef A. H., Albino C. C. (2006). Investigation of the Tolerability of Oral Stevioside in Brazilian Hyperlipidemic Patients. *Brazilian Archives of Biology and Technology*.

[48] Gupta E., Purwar S., Singh A., Sundaram S., Rai G., Sundaram S. (2015). Evaluation of Nutritional, Anti-Nutritional and Bioactive Compounds in Juice and Powder of *stevia rebaudiana*. *Indian Journal of Natural Sciences*.

[49] Sharma S., Walia S., Singh B., Kumar R. (2016). Comprehensive Review on Agro Technologies of Low-Calorie Natural Sweetener Stevia (*Stevia rebaudiana* Bertoni): A Boon to Diabetic Patients. *Journal of the Science of Food and Agriculture*.

[50] Madan S., Ahmad S., Singh G. (2010). *Stevia rebaudiana* (Bert.) Bertoni-a Review. *Indian Journal of Natural Products & Resources*.

[51] Bondarev N., Reshetnyak O., Nosov A. (2001). Peculiarities of Diterpenoid Steviol Glycoside Production in in Vitro Cultures of, *Stevia rebaudiana*, Bertoni. *Plant Science*.

[52] Rafiq M., Dahot M. U., Mangrio S. M., Naqvi H. A., Qarshi I. A. (2007). In Vitro Clonal Propagation and Biochemical Analysis of Field Established, *Stevia rebaudiana*, Bertoni. *Pakistan Journal of Botany*.

[53] Gupta E., Purwar S., Sundaram S., Rai G. (2013). Nutritional and Therapeutic Values of *Stevia rebaudiana*: A Review. *Journal of Medicinal Plants Research*.

[54] Al-Habsi N., Al-Khalili M., Haque S. A., Elias M., Olqi N. A., Al Uraimi T. (2024). Health Benefits of Prebiotics, Probiotics, Synbiotics, and Postbiotics. *Nutrients*.

[55] Oniszczuk A., Oniszczuk T., Gancarz M., Szymańska J. (2021). Role of Gut Microbiota, Probiotics and Prebiotics in the Cardiovascular Diseases. *Molecules*.

[56] Agyekum A. K., Nyachoti C. M. (2017). Nutritional and Metabolic Consequences of Feeding High-Fiber Diets to Swine: A Review. *Engineering*.

[57] Davison K. M., Temple N. J. (2018). Cereal Fiber, Fruit Fiber, and Type 2 Diabetes: Explaining the Paradox. *Journal of Diabetes and its Complications*.

[58] Jha R., Berrocoso J. F. D. (2016). Dietary Fiber and Protein Fermentation in the Intestine of Swine and Their Interactive Effects on Gut Health and on the Environment: A Review. *Animal Feed Science and Technology*.

[59] Sánchez-Muniz F. J. (2012). Dietary Fibre and Cardiovascular Health. *Nutricion Hospitalaria*.

[60] Segura-Campos M., Barbosa-Martín E., Matus-Basto Á. (2014). Comparison of Chemical and Functional Properties of *Stevia rebaudiana* (Bertoni) Varieties Cultivated in Mexican Southeast. *American Journal of Plant Sciences*.

[61] Allam A.-W. I., Nassar A. M., Besheit S. Y. (2001). Nitrogen Fertilizer Requirements of *Stevia rebaudiana* Bertoni, Under Egyptian Conditions. *Egyptian Journal of Agricultural Research*.

[62] Delzenne N. M., Daubioul C., Neyrinck A., Lasa M., Taper H. S. (2002). Inulin and Oligofructose Modulate Lipid Metabolism in Animals: Review of Biochemical Events and Future Prospects. *British Journal of Nutrition*.

[63] Luo J., Van Yperselle M., Rizkalla S. W., Rossi F., Slama G., Bornet F. R. J. (2000). Chronic Consumption of Short-Chain Fructooligosaccharides Does not Affect Basal Hepatic Glucose Production or Insulin Resistance in Type 2 Diabetics. *The Journal of Nutrition*.

[64] Slavin J. (2013). Fiber and Prebiotics: Mechanisms and Health Benefits. *Nutrients*.

[65] Gibson G. R., Hutkins R., Sanders M. E. (2017). Expert Consensus Document: The International Scientific Association for Probiotics and Prebiotics (ISAPP) Consensus Statement on the Definition and Scope of Prebiotics. *Nature Reviews Gastroenterology & Hepatology*.

[66] Koh A., De Vadder F., Kovatcheva-Datchary P., Bäckhed F. (2016). From Dietary Fiber to Host Physiology: Short-Chain Fatty Acids as Key Bacterial Metabolites. *Cell*.

[67] Lemus-Mondaca R., Vega-Gálvez A., Zura-Bravo L., Ah-Hen K. (2012). *Stevia rebaudiana*, Bertoni, Source of a High-Potency Natural Sweetener: A Comprehensive Review on the Biochemical, Nutritional and Functional Aspects. *Food Chemistry*.

[68] Khiraoui A., Bakha M., Amchra F. (2017). Nutritional and Biochemical Properties of Natural Sweeteners of Six Cultivars of, *Stevia rebaudiana*, Bertoni Leaves Grown in Morocco. *Journal of Materials and Environmental Science*.

[69] Gasmalla M. A. A., Yang R., Amadou I., Hua X. (2014). Nutritional Composition of, *Stevia rebaudiana*, Bertoni Leaf: Effect of Drying Method. *Tropical Journal of Pharmaceutical Research*.

[70] Putnik P., Bezuk I., Barba F. J., Lorenzo J. M., Polunić I., Kovačević Bursać D., Barba F. J., Putnik P., Kovačević D. B. (2020). Chapter 5 - Sugar Reduction: *Stevia rebaudiana*, Bertoni as a Natural Sweetener. *Agri-Food Industry Strategies for Healthy Diets and Sustainability*.

[71] Kim I.-S., Yang M., Lee O.-H., Kang S.-N. (2011). The Antioxidant Activity and the Bioactive Compound Content of *Stevia rebaudiana* Water Extracts. *LWT—Food Science and Technology*.

[72] Zubr J. (2010). Carbohydrates, Vitamins and Minerals of *Camelina sativa* Seed. *Nutrition & Food Science*.

[73] Boonkaewwan C., Toskulkao C., Vongsakul M. (2006). Anti-Inflammatory and Immunomodulatory Activities of Stevioside and Its Metabolite Steviol on THP-1 Cells. *Journal of Agricultural and Food Chemistry*.

[74] Kim J.-H., Kang J.-C. (2017). Effects of Sub-Chronic Exposure to Lead (Pb) and Ascorbic Acid in Juvenile Rockfish: Antioxidant Responses, MT Gene Expression, and Neurotransmitters. *Chemosphere*.

[75] Ciji A., Akhtar M. S. (2021). Stress Management in Aquaculture: A Review of Dietary Interventions. *Reviews in Aquaculture*.

[76] Bernal J., Mendiola J. A., Ibáñez E., Cifuentes A. (2011). Advanced Analysis of Nutraceuticals. *Journal of Pharmaceutical and Biomedical Analysis*.

[77] Nasar M. F., Shah S. Z. H., Aftab K., Fatima M., Bilal M., Hussain M. (2021). Dietary Vitamin C Requirement of Juvenile Grass Carp (*Ctenopharyngodon idella*) and Its Effects on Growth Attributes, Organ Indices, Whole-Body Composition and Biochemical Parameters. *Aquaculture Nutrition*.

[78] Pullar J. M., Carr A. C., Vissers M. C. M. (2017). The Roles of Vitamin C in Skin Health. *Nutrients*.

[79] Collins N. (2013). Nutrition 411: Revisiting Vitamin C and Wound Healing. *Ostomy/Wound Management*.

[80] Abdullah M., Jamil R. T., Attia F. N. (2023). *Vitamin C (Ascorbic Acid), StatPearls [Internet]*.

[81] Ibrahim R. E., Amer S. A., Farroh K. Y. (2021). The Effects of Chitosan-Vitamin C Nanocomposite Supplementation on the Growth Performance, Antioxidant Status, Immune Response, and Disease Resistance of Nile Tilapia (*Oreochromis niloticus*) Fingerlings. *Aquaculture*.

[82] Zhang M., Kuang S., Sun Y. (2022). Effects of Dietary Vitamin C on Growth, Antioxidant Enzyme Activity and Immune-Related Gene Expression of *Pampus argenteus*. *Aquaculture Research*.

[83] Ghalibaf M. H. E., Kianian F., Beigoli S. (2023). The Effects of Vitamin C on Respiratory, Allergic and Immunological Diseases: An Experimental and Clinical-Based Review. *Inflammopharmacology*.

[84] Dabrowski K., Ciereszko A. (2001). Ascorbic Acid and Reproduction in Fish: Endocrine Regulation and Gamete Quality. *Aquaculture Research*.

[85] NRC (2011). *Nutrient Requirements of Fish and Shrimp*.

[86] Marcinek K., Krejpcio Z. (2015). *Stevia rebaudiana*, Bertoni—Chemical Composition and Functional Properties. *Acta Scientiarum Polonorum Technologia Alimentaria*.

[87] Pfiffner J. J., Calkins D. G., Bloom E. S., O’Dell B. L. (1946). On the Peptide Nature of Vitamin Bc Conjugate From Yeast. *Journal of the American Chemical Society*.

[88] Darwish A. M. G., Soliman T. N., Elhendy H. A., El-Kholy W. M. (2021). Nano-Encapsulated Iron and Folic Acid-Fortified Functional Yogurt Enhance Anemia in Albino Rats. *Frontiers in Nutrition*.

[89] Lall S. P., Dumas A., Davis D. A. (2022). 3—Nutritional Requirements of Cultured Fish: Formulating Nutritionally Adequate Feeds. *Feed and Feeding Practices in Aquaculture*.

[90] Wolf L. E. (1951). Diet Experiments With Trout: 3. Effect of the Lack of Certain Vitamins on Rainbow Trout. *The Progressive Fish-Culturist*.

[91] Shulpekova Y., Nechaev V., Kardasheva S. (2021). The Concept of Folic Acid in Health and Disease. *Molecules*.

[92] Freisheim J. H., Huennekens F. M. (1969). Folic Acid Coenzymes and One-Carbon Metabolism. XXV. Effect of N-Bromosuccinimide on Dihydrofolate Reductase. *Biochemistry*.

[93] Halver J. E., Halver J. E., Hardy R. W. (2003). 2—The Vitamins. *Fish Nutrition*.

[94] Crider K. S., Yang T. P., Berry R. J., Bailey L. B. (2012). Folate and DNA Methylation: A Review of Molecular Mechanisms and the Evidence for Folate’s Role. *Advances in Nutrition*.

[95] Shiau S.-Y., Lin Y.-H., Lee C.-S., Lim C., Gatlin D. M., Webster C. D. (2015). Vitamins (Excluding C and E). *Dietary Nutrients, Additives, and Fish Health*.

[96] Lin Y.-H., Lin H.-Y., Shiau S.-Y. (2011). Dietary Folic Acid Requirement of Grouper, *Epinephelus malabaricus*, and Its Effects on Non-Specific Immune Responses. *Aquaculture*.

[97] Miao S., Zhang W., Xu W., Mai K. (2013). Dietary Folic Acid Requirement of Juvenile Abalone *Haliotis discus hannai* Ino. *Aquaculture*.

[98] Cowey C. B., Woodward B. (1993). The Dietary Requirement of Young Rainbow Trout (*Oncorhynchus mykiss*) for Folic Acid. *The Journal of Nutrition*.

[99] Lim C., Klesius P. (2001). Influence of Dietary Levels of Folic Acid on Growth Response and Resistance of Nile Tilapia, *Oreochromis niloticus* to *Streptococcus iniae*. *Sixth Asian Fisheries Forum. Book of Abstracts*.

[100] Wanakowat J., Boonyaratpalin M., Pimoljinda T., Assavaaree M. (1989). Vitamin B6 Requirement of Juvenile Seabass *Lates calcarifer*. *The Current Status of Fish Nutrition in Aquaculture*.

[101] Asaikkutti A., Bhavan P. S., Vimala K. (2016). Effects of Different Levels of Dietary Folic Acid on the Growth Performance, Muscle Composition, Immune Response and Antioxidant Capacity of Freshwater Prawn, *Macrobrachium rosenbergii*. *Aquaculture*.

[102] Kar A., Choudhary B. K., Bandyopadhyay N. G. (1999). Preliminary Studies on the Inorganic Constituents of Some Indigenous Hypoglycaemic Herbs on Oral Glucose Tolerance Test. *Journal of Ethnopharmacology*.

[103] Wu S.-J., Ng L.-T., Huang Y.-M. (2005). Antioxidant Activities of *Physalis peruviana*. *Biological and Pharmaceutical Bulletin*.

[104] Lemus-Mondaca R., Ah-Hen K., Vega-Gálvez A., Honores C., Moraga N. O. (2016). *Stevia rebaudiana*, Leaves: Effect of Drying Process Temperature on Bioactive Components, Antioxidant Capacity and Natural Sweeteners. *Plant Foods for Human Nutrition*.

[105] Wölwer-Rieck U. (2012). The Leaves of *Stevia rebaudiana* (Bertoni), Their Constituents and the Analyses Thereof: A Review. *Journal of Agricultural and Food Chemistry*.

[106] Milani P. G., Formigoni M., Lima Y. C. (2017). Fortification of the Whey Protein Isolate Antioxidant and Antidiabetic Activity With Fraction Rich in Phenolic Compounds Obtained From *Stevia rebaudiana* (Bert.). Bertoni Leaves. *Journal of Food Science and Technology*.

[107] Breslin A. (2015). The Chemical Composition of Green Plants, Pharmacotoxic Characterization of the Aqueous Extract From Pereskiagrandifolia Leaves. *Journal of Medicinal Plants Research*.

[108] Molyneux R. J., Lee S. T., Gardner D. R., Panter K. E., James L. F. (2007). Phytochemicals: The Good, the Bad and the Ugly?. *Phytochemistry*.

[109] Guan R., Van Le Q., Yang H. (2021). A Review of Dietary Phytochemicals and Their Relation to Oxidative Stress and Human Diseases. *Chemosphere*.

[110] Chakraborty S. B., Hancz C. (2011). Application of Phytochemicals as Immunostimulant, Antipathogenic and Antistress Agents in Finfish Culture. *Reviews in Aquaculture*.

[111] Chakraborty S. B., Horn P., Hancz C. (2014). Application of Phytochemicals as Growth-Promoters and Endocrine Modulators in Fish Culture. *Reviews in Aquaculture*.

[112] Reverter M., Bontemps N., Lecchini D., Banaigs B., Sasal P. (2014). Use of Plant Extracts in Fish Aquaculture as an Alternative to Chemotherapy: Current Status and Future Perspectives. *Aquaculture*.

[113] Prakash I., Markosyan A., Bunders C. (2014). Development of Next Generation Stevia Sweetener: Rebaudioside M. *Foods*.

[114] Šic Žlabur J., Voća S., Dobričević N., Ježek D., Bosiljkov T., Brnčić M. (2013). *Stevia rebaudiana*, Bertoni-A Review of Nutritional and Biochemical Properties of Natural Sweetener. *Agriculturae Conspectus Scientificus*.

[115] Carakostas M., Prakash I., Kinghorn A. D., Wu C. D., Soejarto D. D. (2012). Steviol Glycosides. *Alternative Sweeteners*.

[116] Zaidan L. B., Dietrich S. M., Felippe G. (1980). Effect of Photoperiod on Flowering and Stevioside Content in Plants of, *Stevia rebaudiana*, Bertoni. *Japanese Journal of Crop Science*.

[117] Puri M., Sharma D., Tiwari A. K. (2011). Downstream Processing of Stevioside and Its Potential Applications. *Biotechnology Advances*.

[118] Casas-Grajales S., Ramos-Tovar E., Chávez-Estrada E. (2019). Antioxidant and Immunomodulatory Activity Induced by Stevioside in Liver Damage: In vivo, in Vitro and in Silico Assays. *Life Sciences*.

[119] Daneshyar M., Geuns J. M. C., Willemsen H. (2012). Evaluation of Dietary Stevioside Supplementation on Anti-Human Serum Albumin Immunoglobulin G, Alpha-1-Glycoprotein, Body Weight and Thyroid Hormones in Broiler Chickens. *Journal of Animal Physiology and Animal Nutrition*.

[120] Chen K. (2002). Effects of Stevioside Solution and Its pH on the Feeding Behavior of Yellow Catfish (*Pelteobagarus fulvidraco* Richardson). *Journal of Southwest Agricultural University*.

[121] Heidari N., Chelehmal M., Javaheri Baboli M. (2018). The Effect of Adding Stevia (*Stevia rebaudian*) Extract in the Diet on the Growth, Survival and Fillet Chemical Properties on Common Carp (*Cyprinus carpio*). *Applied Biology*.

[122] Koyama E., Kitazawa K., Ohori Y. (2003). In Vitro Metabolism of the Glycosidic Sweeteners, Stevia Mixture and Enzymatically Modified Stevia in Human Intestinal Microflora. *Food and Chemical Toxicology*.

[123] Abasubong K. P., Li X.-F., Adjoumani J.-J. Y., Jiang G.-Z., Desouky H. E., Liu W.-B. (2022). Effects of Dietary Xylooligosaccharide Prebiotic Supplementation on Growth, Antioxidant and Intestinal Immune-Related Genes Expression in Common Carp *Cyprinus carpio* Fed a High-Fat Diet. *Journal of Animal Physiology and Animal Nutrition*.

[124] Masumura T., Sugahara M., Noguchi T., Mori K., Naito H. (1985). The Effect of Gizzerosine, a Recently Discovered Compound in Overheated Fish Meal, on the Gastric Acid Secretion in Chicken. *Poultry Science*.

[125] Fu X., Liu Y., Zhou H. (2023). Effects of Dietary Histamine on the Growth, Digestive Tract Morphology, Flesh Quality in Striped Catfish. *Pangasianodon Hypophthalmus*.

[126] Watanabe T., Takeuchi T., Satoh S., Toyama K., Okuzumi M. (1987). Effect of Dietary Histidine or Histamine on Growth and Development of Stomach Erosion in Rainbow Trout. *NIPPON SUISAN GAKKAISHI*.

[127] Shiozaki K., Fujii A., Nakano T., Yamaguchi T., Sato M. (2006). Inhibitory Effects of Hot Water Extract of the Stevia Stem on the Contractile Response of the Smooth Muscle of the Guinea Pig Ileum. *Bioscience, Biotechnology, and Biochemistry*.

[128] Choy J., Romano N., Ebrahimi M., Kamarudin M. S. (2018). Effects of Dietary Steviol Glycosides on the Growth, Feed Intake and Intestinal Short-Chain Fatty Acids in Red Hybrid Tilapia. *Journal of Environmental Biology*.

[129] Reyes-Sosa C. F., Castellanos-Molina R. (1995). Nutritional Evaluation of Gizzard Erosion Positive Brown Fish Meal in Starter Diets for Nile Tilapia, *Oreochromis niloticus*. *Aquaculture*.

[130] Dyduch-Siemińska M., Najda A., Gawroński J. (2020). Bertoni, a Source of High-Potency Natural Sweetener-Biochemical and Genetic Characterization. *Molecules*.

[131] Kroyer G. (2010). Stevioside and Stevia-Sweetener in Food: Application, Stability and Interaction With Food Ingredients. *Journal für Verbraucherschutz Und Lebensmittelsicherheit*.

[132] Nakayama K., Kasahara D., Yamamoto F. (1986). Absorption, Distribution, Metabolism and Excretion of Stevioside in Rats. *Food Hygiene and Safety Science (Shokuhin Eiseigaku Zasshi)*.

[133] Geuns J. M. C., Buyse J., Vankeirsbilck A., Temme E. H. M. (2007). Metabolism of Stevioside by Healthy Subjects. *Experimental Biology and Medicine*.

[134] Hahor W., Thongprajukaew K., Suanyuk N. (2019). Effects of Dietary Supplementation of Oligosaccharides on Growth Performance, Gut Health and Immune Response of Hybrid Catfish (*Pangasianodon gigas* × *Pangasianodon hypophthalmus*). *Aquaculture*.

[135] Hellfritsch C., Brockhoff A., Stähler F., Meyerhof W., Hofmann T. (2012). Human Psychometric and Taste Receptor Responses to Steviol Glycosides. *Journal of Agricultural and Food Chemistry*.

[136] Suckling J., Morse S., Murphy R. (2023). Environmental Life Cycle Assessment of Production of the High Intensity Sweetener Steviol Glycosides From *Stevia rebaudiana* Leaf Grown in Europe: The SWEET Project. *The International Journal of Life Cycle Assessment*.

[137] Cardello H. M. A. B., Da Silva M. A. P. A., Damasio M. H. (1999). Measurement of the Relative Sweetness of Stevia Extract, Aspartame and Cyclamate/Saccharin Blend as Compared to Sucrose at Different Concentrations. *Plant Foods for Human Nutrition*.

[138] Sánchez-Aceves L. M., Dublán-García O., López-Martínez L.-X. (2017). Reduction of the Oxidative Stress Status Using Steviol Glycosides in a Fish Model (*Cyprinus carpio*). *BioMed Research International*.

[139] Harada K., Miyasaki T., Tamura Y. (1994). Chemoattractant Effects of Sugars and Their Related Compounds on Black Abalone *Haliotis discus*. *Comparative Biochemistry and Physiology Part A: Physiology*.

[140] Harada K., Miyasaki T. (1997). Attractivity of Exotic Fruit Fleshes for the Abalone, the Oriental Weatherfish, and the Yellowtail. *Fisheries Science*.

[141] Wang L. S., Shi Z., Shi B. M., Shan A. S. (2014). Effects of Dietary Stevioside/Rebaudioside A on the Growth Performance and Diarrhea Incidence of Weaned Piglets. *Animal Feed Science and Technology*.

[142] Muanda F. N., Soulimani R., Diop B., Dicko A. (2011). Study on Chemical Composition and Biological Activities of Essential Oil and Extracts From *Stevia rebaudiana* Bertoni Leaves. *LWT - Food Science and Technology*.

[143] Shukla S., Mehta A., Bajpai V. K., Shukla S. (2009). In Vitro Antioxidant Activity and Total Phenolic Content of Ethanolic Leaf Extract of, *Stevia rebaudiana*, Bert. *Food and Chemical Toxicology*.

[144] Tadhani M. B., Patel V. H., Subhash R. (2007). In Vitro Antioxidant Activities of *Stevia rebaudiana* Leaves and Callus. *Journal of Food Composition and Analysis*.

[145] Heleno S. A., Martins A., Queiroz M. J. R. P., Ferreira I. C. F. R. (2015). Bioactivity of Phenolic Acids: Metabolites versus Parent Compounds: A Review. *Food Chemistry*.

[146] Rice-Evans C. A., Miller N. J., Paganga G. (1996). Structure-Antioxidant Activity Relationships of Flavonoids and Phenolic Acids. *Free Radical Biology and Medicine*.

[147] Lemus-Mondaca R., Vega-Gálvez A., Rojas P. (2018). Antioxidant, Antimicrobial and Anti-Inflammatory Potential of *Stevia rebaudiana* Leaves: Effect of Different Drying Methods. *Journal of Applied Research on Medicinal and Aromatic Plants*.

[148] Pacifico S., Piccolella S., Nocera P., Tranquillo E., Dal Poggetto F., Catauro M. (2019). New Insights into Phenol and Polyphenol Composition of *Stevia rebaudiana* Leaves. *Journal of Pharmaceutical and Biomedical Analysis*.

[149] Yu H., Yang G., Sato M., Yamaguchi T., Nakano T., Xi Y. (2017). Antioxidant Activities of Aqueous Extract From *Stevia rebaudiana* Stem Waste to Inhibit Fish Oil Oxidation and Identification of Its Phenolic Compounds. *Food Chemistry*.

[150] Zhang Q., Yang H., Li Y., Liu H., Jia X. (2017). Toxicological Evaluation of Ethanolic Extract From, *Stevia rebaudiana*, Bertoni Leaves: Genotoxicity and Subchronic Oral Toxicity. *Regulatory Toxicology and Pharmacology*.

[151] Myint K. z., Wu K., Xia Y. (2020). Polyphenols From *Stevia rebaudiana* (Bertoni) Leaves and Their Functional Properties. *Journal of Food Science*.

[152] Abasubong K. P., Gabriel N. N., Adjoumani J.-J. Y., Gabriel N. N., Omoregie E., Abasubong K. P. (2023). Ferulic Acid as Feed Additives in Aquaculture: A Review on Growth, Immune Response, and Antioxidant Status of Finfish. *Emerging Sustainable Aquaculture Innovations in Africa*.

[153] Gullón B., Gagaoua M., Barba F. J., Gullón P., Zhang W., Lorenzo J. M. (2020). Seaweeds as Promising Resource of Bioactive Compounds: Overview of Novel Extraction Strategies and Design of Tailored Meat Products. *Trends in Food Science & Technology*.

[154] Garg S. K., Shukla A., Choudhury S., Gupta R. C., Srivastava A., Lall R. (2019). Polyphenols and Flavonoids. *Nutraceuticals in Veterinary Medicine*.

[155] Jan R., Asaf S., Numan M., Lubna, Kim K.-M. (2021). Plant Secondary Metabolite Biosynthesis and Transcriptional Regulation in Response to Biotic and Abiotic Stress Conditions. *Agronomy*.

[156] Falcone Ferreyra M. L., Rius S. P., Casati P. (2012). Flavonoids: Biosynthesis, Biological Functions, and Biotechnological Applications. *Frontiers in Plant Science*.

[157] Yao L. H., Jiang Y. M., Shi J. (2004). Flavonoids in Food and Their Health Benefits. *Plant Foods for Human Nutrition*.

[158] de Souza Farias S. A., da Costa K. S., Martins J. B. L. (2021). Analysis of Conformational, Structural, Magnetic, and Electronic Properties Related to Antioxidant Activity: Revisiting Flavan, Anthocyanidin, Flavanone, Flavonol, Isoflavone, Flavone, and Flavan-3-ol. *ACS Omega*.

[159] Batiha G. E.-S., Beshbishy A. M., Ikram M. (2020). The Pharmacological Activity, Biochemical Properties, and Pharmacokinetics of the Major Natural Polyphenolic Flavonoid: Quercetin. *Foods*.

[160] Ferreyra M. L. F., Serra P., Casati P. (2021). Recent Advances on the Roles of Flavonoids as Plant Protective Molecules after UV and High Light Exposure. *Physiologia Plantarum*.

[161] Middleton E., Kandaswami C., Theoharides T. C. (2000). The Effects of Plant Flavonoids on Mammalian Cells: Implications for Inflammation, Heart Disease, and Cancer. *Pharmacological Reviews*.

[162] Boots A. W., Haenen G. R. M. M., Bast A. (2008). Health Effects of Quercetin: From Antioxidant to Nutraceutical. *European Journal of Pharmacology*.

[163] Armobin K., Ahmadifar E., Adineh H. (2023). Quercetin Application for Common Carp (*Cyprinus carpio*): I. Effects on Growth Performance, Humoral Immunity, Antioxidant Status, Immune-Related Genes, and Resistance against Heat Stress. *Aquaculture Nutrition*.

[164] Chen Z., Fan D., Pan L., Su C., Ding Y., Lu M. (2023). Study of Effects of Dietary Quercetin (Que) on Growth Performance and Disease Resistance Mechanism of *Litopenaeus vannamei*. *Aquaculture*.

[165] Ghanta S., Banerjee A., Poddar A., Chattopadhyay S. (2007). Oxidative DNA Damage Preventive Activity and Antioxidant Potential of *Stevia rebaudiana* (Bertoni) Bertoni, a Natural Sweetener. *Journal of Agricultural and Food Chemistry*.

[166] Jan R., Khan M., Asaf S., Lubna, Asif S., Kim K.-M. (2022). Bioactivity and Therapeutic Potential of Kaempferol and Quercetin: New Insights for Plant and Human Health. *Plants*.

[167] Nirmala P., Ramanathan M. (2011). Effect of Kaempferol on Lipid Peroxidation and Antioxidant Status in 1,2-Dimethyl Hydrazine Induced Colorectal Carcinoma in Rats. *European Journal of Pharmacology*.

[168] McLaughlin J., Hostettmann K. (1991). Methods in Plant Biochemistry. *Assays for Bioactivity*.

[169] Smeriglio A., Barreca D., Bellocco E., Trombetta D. (2017). Proanthocyanidins and Hydrolysable Tannins: Occurrence, Dietary Intake and Pharmacological Effects. *British Journal of Pharmacology*.

[170] Mueller-Harvey I. (2001). Analysis of Hydrolysable Tannins. *Animal Feed Science and Technology*.

[171] Gyamfi M. A., Aniya Y. (2002). Antioxidant Properties of Thonningianin A, Isolated From the African Medicinal Herb, *Thonningia sanguinea*. *Biochemical Pharmacology*.

[172] Serrano J., Puupponen-Pimiä R., Dauer A., Aura A.-M., Saura-Calixto F. (2009). Tannins: Current Knowledge of Food Sources, Intake, Bioavailability and Biological Effects. *Molecular Nutrition & Food Research*.

[173] Zhang L., Guan Q., Jiang J., Khan M. S. (2023). Tannin Complexation With Metal Ions and Its Implication on Human Health, Environment and Industry: An Overview. *International Journal of Biological Macromolecules*.

[174] Gershenzon J., Dudareva N. (2007). The Function of Terpene Natural Products in the Natural World. *Nature Chemical Biology*.

[175] Ramya R., Punitha S. C., Aruna G. (2021). Analysis of Bioactive Compounds From, *Stevia rebaudiana*, Bertoni. *Nveo-Natural Volatiles & Essential Oils Journal| NVEO*.

[176] Kim Y., Lounds-Singleton A. J., Talcott S. T. (2009). Antioxidant Phytochemical and Quality Changes Associated With Hot Water Immersion Treatment of Mangoes (*Mangifera indica* L.). *Food Chemistry*.

[177] Blanco G., Sánchez B., Fdez-Riverola F., Margolles A., Lourenço A. (2019). In Silico Approach for Unveiling the Glycoside Hydrolase Activities in *Faecalibacterium prausnitzii* Through a Systematic and Integrative Large-Scale Analysis. *Frontiers in Microbiology*.

[178] Purkayastha S., Kwok D. (2020). Metabolic Fate in Adult and Pediatric Population of Steviol Glycosides Produced From Stevia Leaf Extract by Different Production Technologies. *Regulatory Toxicology and Pharmacology*.

[179] Wen K., Zhang K., Gao W. (2024). Effects of Stevia Extract on Production Performance, Serum Biochemistry, Antioxidant Capacity, and Gut Health of Laying Hens. *Poultry Science*.

[180] Khalifa A., El-Saadany A., Hassan M., Kashyout W., Dosoky W. (2021). Impact of Stevioside Supplementation as Feed Additive in Finisher Broiler Diets on Growth Performance, Carcass Traits, Meat Quality, Selected Biochemical Parameters, and Caecum Microflora. *Advances in Animal and Veterinary Sciences*.

[181] Atteh J. O., Onagbesan O. M., Tona K., Decuypere E., Geuns J. M. C., Buyse J. (2008). Evaluation of Supplementary Stevia (*Stevia rebaudiana*, Bertoni) Leaves and Stevioside in Broiler Diets: Effects on Feed Intake, Nutrient Metabolism, Blood Parameters and Growth Performance. *Journal of Animal Physiology and Animal Nutrition*.

[182] Xu L., Wei Y., Wang J. (2021). Effects of the Natural Sweetener Rebaudioside A on Intake, Nutrient Digestion, Rumen Fermentation, and Blood Biochemical Parameters in Adult Goats During Summer. *Animal Science Journal*.

[183] Awney H. A., Massoud M. I., El-Maghrabi S. (2011). Long-Term Feeding Effects of Stevioside Sweetener on Some Toxicological Parameters of Growing Male Rats. *Journal of Applied Toxicology*.

[184] Jiang J., Liu S., Jamal T. (2020). Effects of Dietary Sweeteners Supplementation on Growth Performance, Serum Biochemicals, and Jejunal Physiological Functions of Broiler Chickens. *Poultry Science*.

[185] Jeppesen P. B., Dyrskog S. E., Agger A. (2006). Can Stevioside in Combination With a Soy-Based Dietary Supplement Be a New Useful Treatment of Type 2 diabetes? An in Vivo Study in the Diabetic Goto-Kakizaki Rat. *The Review of Diabetic Studies*.

